# Milk proteins as mastitis markers in dairy ruminants - a systematic review

**DOI:** 10.1007/s11259-022-09901-y

**Published:** 2022-02-23

**Authors:** Anna Giagu, Martina Penati, Sara Traini, Simone Dore, Maria Filippa Addis

**Affiliations:** 1 Dipartimento di Medicina Veterinaria, Università di Sassari, Sassari, Italy; 2 Istituto Zooprofilattico Sperimentale della Sardegna, Centro di Referenza Nazionale per le Mastopatie degli Ovini e dei Caprini, Sassari, Italy; 3 ARES Sardegna, ASL, Nuoro, Italy; 4 Dipartimento di Medicina Veterinaria e Scienze Animali, Università di Milano, Lodi, Italy

**Keywords:** immunoassay, intramammary infection, amyloid A, haptoglobin, cathelicidin

## Abstract

Mastitis is one of the most impacting diseases in dairy farming, and its sensitive and specific detection is therefore of the greatest importance. The clinical evaluation of udder and mammary secretions is typically combined with the milk Somatic Cell Count (SCC) and often accompanied by its bacteriological culture to identify the causative microorganism. In a constant search for improvement, several non-enzymatic milk proteins, including milk amyloid A (M-SAA), haptoglobin (HP), cathelicidin (CATH), and lactoferrin (LF), have been investigated as alternative biomarkers of mastitis for their relationship with mammary gland inflammation, and immunoassay techniques have been developed for detection with varying degrees of success. To provide a general overview of their implementation in the different dairy species, we carried out a systematic review of the scientific literature using the PRISMA (Preferred Reporting Items for Systematic Reviews and Meta-Analysis) guidelines. Our review question falls within the type *“Diagnostic test accuracy questions”* and aims at answering the diagnostic question: “*Which are the diagnostic performances of mastitis protein biomarkers investigated by immunoassays in ruminant milk?”*. Based on 13 keywords combined into 42 searches, 523 manuscripts were extracted from three scientific databases. Of these, 33 passed the duplicate removal, title, abstract, and full-text screening for conformity to the review question and document type: 78.8% investigated cows, 12.1% sheep, 9.1% goats, and 6.1% buffaloes (some included more than one dairy species). The most frequently mentioned protein was M-SAA (48.5%), followed by HP (27.3%), CATH (24.2%) and LF (21.2%). However, the large amount of heterogeneity among studies in terms of animal selection criteria (45.5%), index test (87.9%), and standard reference test (27.3%) resulted in a collection of data not amenable to meta-analysis, a common finding illustrating how important it is for case definitions and other criteria to be standardized between studies. Therefore, results are presented according to the SWiM (Synthesis Without Meta-analysis) guidelines. We summarize the main findings reported in the 33 selected articles for the different markers and report their results in form of comparative tables including sample selection criteria, marker values, and diagnostic performances, where available. Finally, we report the study limitations and bias assessment findings.

**Supplementary Information:**

The online version contains supplementary material available at 10.1007/s11259-022-09901-y.

## Introduction

As a critical factor affecting milk yield and quality, mastitis represents the most relevant health problem in dairy ruminants worldwide (Ruegg 2017). According to the National Mastitis Council (NMC) mastitis is defined as *“an inflammation of one or more quarters/halves of the mammary gland, almost always caused by an infecting microorganism”* (Lopez-Benavides et al. 2012). Whereas clinical mastitis can be diagnosed by examination of the udder and of the milk for visible abnormalities, identifying subclinical mastitis is more challenging (Menzies and Ramanoon 2001; Oliver et al. 2004). In animals with subclinical mastitis, the diagnosis is mainly performed on the milk through indirect methods such as the Somatic Cell Count (SCC) (Bergonier et al. 2003; Persson and Olofsson 2011) or its field version, the California Mastitis Test (CMT) (Kelly et al. 2018). Being typically caused by an intramammary infection (IMI) (Ezzat Alnakip et al. 2014), the disease is also investigated through direct methods such as the bacteriological culture (BC) (Contreras et al. 2007) or molecular assays (i.e., PCR) (Chakraborty et al. 2019). The indirect screening approaches rely mainly on the principle that the udder microenvironment changes during the inflammatory process, with an increase in the concentration of immune cells and immune mediators (Hughes and Watson 2018). Polymorphonuclear neutrophils (PMNs) are the prevalent immune cells in the acute phase of mastitis; therefore, SCC and CMT perform well as diagnostic tools because of their indirect relationship to the presence of PMNs (Leitner et al. 2000; Sordillo and Streicher 2002). However, these tests may lack specificity (Rossi et al. 2018), especially in small ruminants (Souza et al. 2012). On the other hand, BC lacks sensitivity (Chakraborty et al. 2019), and it is hardly applicable as a mastitis screening tool given its requirements in terms of time, labor, and cost. Clinical examination, SCC, CMT, and BC, should be used in combination for increasing diagnostic performance (Lam et al. 2009; Chakraborty et al. 2019); however, a universally accepted specific diagnostic algorithm or protocol is not yet available.

During mammary gland inflammation, numerous antibacterial and immune defense proteins, including Acute Phase Proteins (APPs), lactoferrin (LF), cathelicidins (CATH), cytokines, chemokines, and growth factors, are released in the milk and can potentially serve as “mastitis markers” (Smolenski et al. 2011; Thomas et al. 2015). Accordingly, their implementation as alternative/integrative diagnostic tools has been the subject of several studies during the last decades (Viguier et al. 2009). Many of them focused on discovering new biomarkers for implementing diagnostic tools with improved sensitivity and specificity when compared to the currently available assays. For inflammation-related proteins devoid of intrinsic enzymatic activity, the measurement methods are typically immunoassays employing highly specific antibodies (Viguier et al. 2009). Adding to the possibility of increased diagnostic performances, the integration of traditional diagnostic approaches with immunoassays measuring mastitis marker proteins might bring additional benefits, including the ability to work efficiently on frozen samples, the high analytical throughput, the relatively low analytical costs, and the minimal requirements for dedicated personnel training, specialized or expensive instrumentations (Addis et al. 2016a).

A group of widely investigated potential biomarkers are Acute Phase Proteins (APPs), commonly employed as clinical biomarkers of inflammation in serum but also found in the milk. In particular, the milk isoforms of serum amyloid A (M-SAA) and haptoglobin (HP) (Hussein et al. 2018; Chakraborty et al. 2019; Iliev and Georgieva 2019;) are among the most employed ones. Other proteins indicated as suitable mastitis markers are lactoferrin (LF) (Shimazaki and Kawai 2017) and cathelicidins (CATH) (Smolenski et al. 2011).

Biomarker discovery and implementation are constantly evolving, and comparative data on their diagnostic performances are lacking. Therefore, it is not easy to establish their relative advantages in the different dairy ruminant species compared to the current diagnostic approaches. To provide an organic overview of the topic, to understand if the data currently available in the literature are amenable to meta-analysis, and to attempt a comparative assessment of the respective diagnostic performances, we carried out a literature survey using the systematic review approach based on the PRISMA (Preferred Reporting Items for Systematic Reviews and Meta-Analyses) guidelines. In veterinary medicine, the methodology for systematic reviews has been defined by Sargeant and O’Connor (2020), who identified specific steps to follow. Accordingly, our review question falls within the fourth type, *“Diagnostic test accuracy questions”*, aimed at summarizing diagnostic test accuracy. Specifically, this systematic review aims at examining the scientific literature to answer the diagnostic question: “*Which are the diagnostic performances of mastitis protein biomarkers investigated by immunoassays in ruminant milk?”*.

## Methods

### Information sources and search strategy

We carried out this systematic review according to the guidelines of the PRISMA statement (Moher et al. 2009). We searched three different databases (i.e., MedLine, Scopus, and Web of Science) until January 28, 2021. For Scopus searches, we applied the default search settings (Article title, abstract, and keywords), whereas in Web of Science we used the specific database “Web of Science Core Collection”. Our review question falls within the fourth type, *“Diagnostic test accuracy questions”*, aimed at summarizing diagnostic test accuracy, and at answering the diagnostic question: “*Which are the diagnostic performances of mastitis protein biomarkers investigated by immunoassays in ruminant milk?”* as suggested by Sargeant and O’Connor (2020) for systematic reviews in veterinary medicine. Accordingly, the search terms included the words “biomarker”, “marker”, “intramammary infection”, “mastitis”, “milk”. These search terms were enriched with the most common markers and detection assays to improve the retrieval of relevant scientific articles. Concerning markers, an initial survey of the literature indicated that the ones most associated with the words “milk” and “mastitis” were M-SAA, HP, LF, and CATH. On the other hand, the two immunoassays most frequently used for measuring protein markers devoid of intrinsic enzymatic activity were ELISA and lateral flow/immunochromatography. Once defined, we combined the search terms and their related Mesh terms into 42 specific searches, as follows: (“biomarker” OR “marker” OR “amyloid” OR “haptoglobin” OR “cathelicidin” OR “lactoferrin”) AND (“intramammary infection” OR “mastitis”) AND (“milk”) AND (“immunoassay” OR “ELISA” OR “lateral flow” OR “immunochromatography”) (Supplementary Table I).

### Study selection, data extraction, and synthesis method

Three researchers (AG, ST, and MP) independently screened title, abstract, and full-text for assessing the article compliance with the review question and solved any disagreement by discussion and consensus. When necessary, a fourth researcher with expertise in the field (MFA) was consulted to reach an exclusion decision. Adding to the articles not relating to the review question, we excluded those written in languages different from English and belonging to the categories review, case report, report, book chapter, editorial, abstract, and letter. From each eligible document, the following data were extracted: species, first author, year, country, study design, biomarker, technique, sample type and size, SCC, pathogens, unit of measurement, results, sensitivity, specificity, and cut-off. To synthesize the results we applied the *“Synthesis Without Meta-analysis”* (SWiM) guidelines (Campbell et al. 2020) by using tables and graphs.

### Quality assessment

The tool consists of 14 questions and two main sections, bias assessment and applicability, including four key domains and three key domains, respectively. In bias assessment, for every study, were assessed the “animal selection” strategy, the “index test”, the “reference standard”, and “flow and timing”. The term "index test" is referred to the test object of study, while "reference standard" to the standard test considered the best available test to diagnose the disease of interest (i.e. a single test, follow-up or combination of tests).

In the applicability assessment, we collected and rated how much the studies matched the review question. For both sections, the risk was expressed as “high”, “low”, and “unclear” risk when data were insufficient. The 33 screened records showed high heterogeneity in study design, animal selection, and standard reference tests.

## Results and discussion

### Results of the PRISMA procedure

**Fig. 1:**
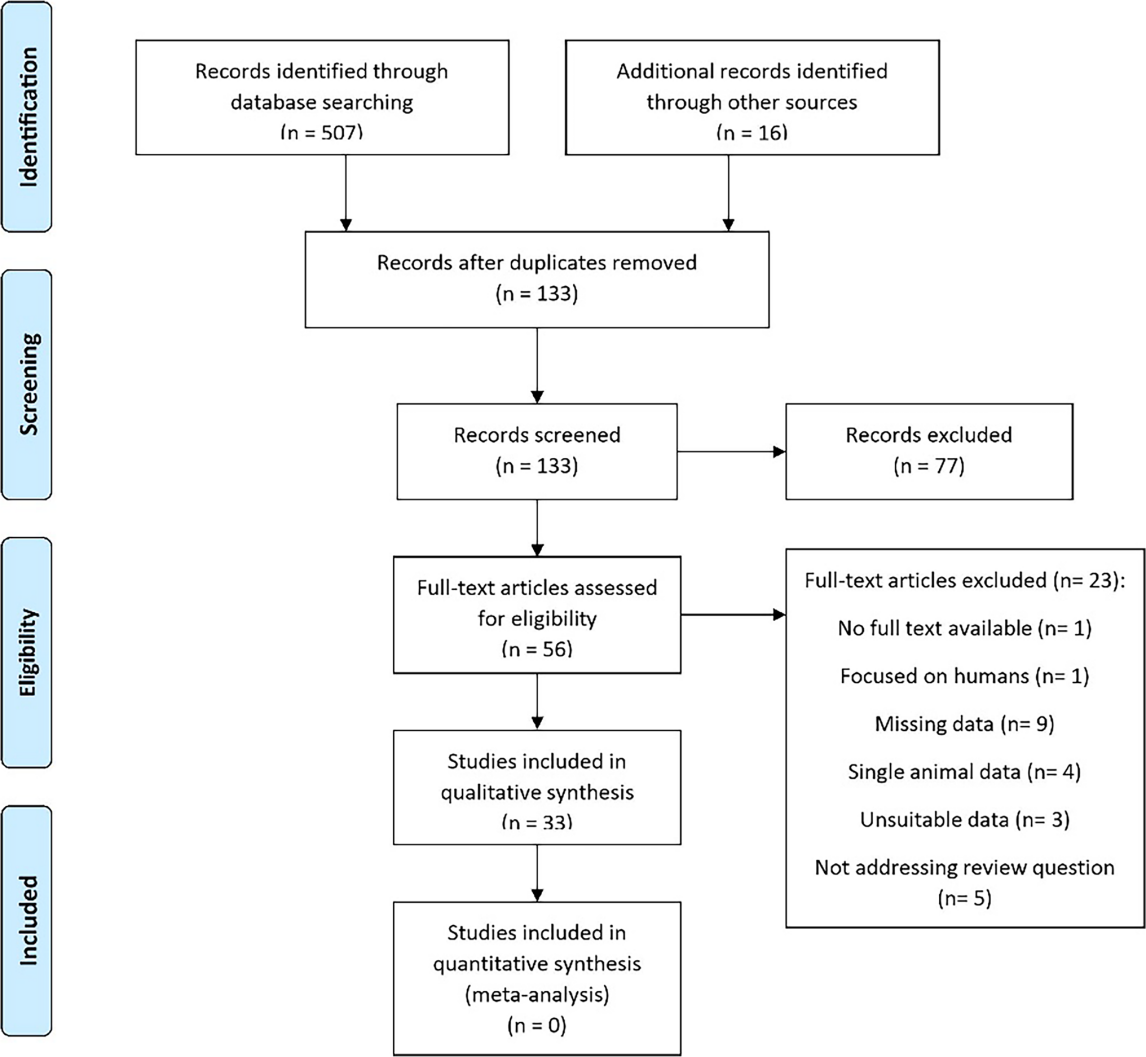
PRISMA 2009 flow diagram

The steps of the literature search are summarized in the PRISMA 2009 flow diagram (Fig. 1). The search led to the identification of 507 scientific papers (220 MedLine + 131 Scopus + 156 Web of Science); 16 further records were then added to the original search through an expert revision of the literature, resulting in 523 manuscripts (Supplementary Tables II, III, IV, and V). After removing duplicates, 133 records entered three main screening steps. Firstly, records were screened on the title, secondly on the abstract (n = 72, intermediate step not included in Fig.1), and finally on the full-text for evaluating the eligibility to qualitative and quantitative analysis (Supplementary Tables VI and VII). As a result of this procedure, 33 scientific articles were considered eligible (Supplementary Table VIII).

### Species overview

By sorting the number of papers based on the dairy species, out of 33 manuscripts, 26 (78.8%) investigated cows, 4 (12.1%) sheep, 3 (9.1%) goats, and 2 (6.1%) buffaloes (Table 1). The total number of records does not match because 2 papers addressed more than one species.

**Table 1:** Species and biomarker overview

Biomarker	%	N°	Cow	Sheep	Buffalo	Goat
M-SAA	48.5	16/33	15	1	-	-
HP	27.3	9/33	9	-	-	-
CATH	24.2	8/33	2	2	1	3
LF	21.2	7/33	5	-	1	1
IL1β	9.1	3/33	2	1	-	-
IL6	9.1	3/33	2	1	-	-
AGP	3	1/33	1	-	-	-
BSA	3	1/33	1	-	-	-
CRP	3	1/33	1	**-**	-	-
IgG	3	1/33	1	-	-	-
IL8	3	1/33	-	1	-	-
IL10	3	1/33	1	-	-	-
IL12	3	1/33	1	-	-	-
LBP	3	1/33	1	-	-	-
TGFα	3	1/33	1	-	-	-
TGFβ	3	1/33	1	-	-	-
TNFα	3	1/33	1	-	-	-

Acronyms: M-SAA, milk amyloid A. HP, haptoglobin. CATH, cathelicidins. LF, lactoferrin. IL1β, interleukin 1β. IL6, interleukin 6. AGP, Alpha-1-Acid Glycoprotein. BSA, bovine serum albumin. CRP, C-reactive protein. IgGs, immunoglobulin G. IL8, interleukin 8. IL10, interleukin 10. IL12, interleukin 12. LBP, lipopolysaccharide-binding protein. TGFα, Transforming Growth Factor α. TGFβ, Transforming Growth Factor β. TNFα, Tumor Necrosis Factor α. %, percent of articles relating to the numbers of papers addressing the biomarker out of the total. N°, number of articles relating to specific biomarker out of the 33 eligible records

***Cow.*** Out of 26 papers on cow milk, 15 (57.7%) investigated M-SAA, 9 (34.6%) HP, 5 (19.2%) LF, 2 CATH (7.7%), interleukin 1β (IL1β) and interleukin 6 (IL-6). Other biomarkers were Alpha-1-Acid Glycoprotein (AGP), bovine serum albumin (BSA), C-reactive protein (CRP), immunoglobulin G (IG), interleukin 8 (IL8), interleukin 10 (IL10), interleukin 12 (IL12) lipopolysaccharide-binding protein (LBP), Transforming Growth Factor α (TGFα), Transforming Growth Factor β (TGFβ), and Tumor Necrosis Factor α (TNFα), and they were addressed in 1 paper each (3.8%) (Table 1). The samples were represented by quarter milk in 18/26 (69.2%), and by composite milk in 8/26 (30.8%). In one record (Sobczuk-Szul et al. 2014), the milk sample type was not specified, whereas in another study (Thomas et al. 2015) both quarter and composite samples were used. Concerning the diagnostic methods, ELISA was used in 25 (96.2%) records, whereas in 1 paper (3.8%) the biomarker was investigated by SPARCL. Moreover, we observed 25 (96.2%) observational studies, related to natural inflammation/infection, and only one experimental infection study. Tables 2, 3, 4 and 5 summarize the main findings of the 26 papers evaluating cows.

**Table 2:** Cow, results obtained for M-SAA by applying ELISA and SPARCL* (Dalanezi et al. 2020). The unit of measurement is μg/mL.

*Study*	SCC (cells/ml x 10^3^)	Pathogens	Sample	Sample group	N	Value	%Se	%Sp	cutoff
Kováč et al. 2007			*Composite*			*Median / Range*			
	<100			SCC group 1	5	0.67 / 0-1.79			
	100-400			SCC group 2	5	1.52 / 0-7.15			
	>400			SCC group 3	8	26.54 / 1.81-54.28			
Suojala et al. 2008			*Quarter*		*7*	*Mean (SD)*			
		*E. coli*		Clinical		1.32 (0.95)			
Åkerstedt et al. 2009	*Mean (SD)*		*Composite*		*68*	*Mean (SD)*			
	218 (179)					1.12 (1.16)			
Gerardi et al. 2009	*Mean / Median*		*Quarter*			*Mean / Median*			
	618 / 163			Subclinical (TP-802)	40	9.8 / 2.3			
				Subclinical (TP-807)		5.5 / 0.5			
	2,704 / 1,120			Clinical (TP-802)	24	16.1 / 8.0			
				Clinical (TP-807)		6.9 / 3.8			
	58 / 28			Healthy (TP-802)	4	0.5 / 0.4			
				Healthy (TP-807)		0.1 / 0.1			
Safi et al. 2009	*Mean (SD) / Median / Range*		*Quarter*			*Mean (SD) / Median / Range*			
	5,000 (9,500) /1,250 / 3-51,840	*S. aureus*			106	67 (120) / 28 / 14-843			
	93 (68)/75 / 3-266	Negative		Healthy	134	9 (5) / 8 / 1-29			
							90.6	98.3	16.4
Pyörälä et al. 2011			*Quarter*			*Median (IQR)*			
				Subclinical	136	13.4 (3-83.5)			
				Clinical	98	22.7 (4.4-102.5)			
		*S. aureus*			44	16.4 (3.5-80.2)			
		NAS			45	4.4 (1.6-70.5)			
		*S. uberis*			43	21.2 (5.2-99.5)			
		*S. dysgalactiae*			48	23.9 (7.7-85.3)			
		*E. coli*			23	279.5 (19.7-675.0)			
		*A. pyogenes*			24	3.0 (<0.3-41.8)			
		Other			7	41.3 (3.9-720.0)			
Kovačević-Filipović et al. 2012	*Mean (SD) /Range*		*Quarter*			*Mean (SD) /Range*			
	20 (2) / 7-31	Negative		Control group	10	1.4 (1.1) / 0-4.15			
	2,066 (28) / 556-3,617	*S. aureus*		Subclinical	10	102.6 (88.8) / 27.9-324.5			
Shirazi-Beheshtiha et al. 2011			*Quarter*			*Mean (SD) / Median / Range*			
	<130	Negative		Healthy	38	0.61 (0.5) / 0.44 / 0.1-1.9			
	>130	Positive		Subclinical	52	12.83 (12.8) / 7.88 / 0.84-50.5			
							92.3	92.1	1.6
Szczubiał et al. 2012			*Quarter*			*Mean (SD) /Range*			
		*S. aureus*		Subclinical	12	41.75 (26.6) / 8.76-74.75			
		*S. agalactiae*			9	60.11 (17.53) / 9.78-111.26			
		*S. dysgalactiae*			18	72.43 (43.22) / 19.90-121.20			
		*S. uberis*			18	92.23 (28.64) / 8.58-221.64			
		NAS			19	12.47 (6.95) / 6.53-23.33			
		*Candida* spp.			8	101.00 (55.56) / 9.61-199.36			
		Negative		Healthy	14	11.67 (7.40) / 5.24-19.04			
Wollowski et al. 2021			*Quarter*			*Mean*			
				Subclinical	107	2.62			
				Clinical (all)	201	6.67			
				Clinical (mild)	45	6.14			
				Clinical (moderate)	95	5.69			
				Clinical (severe)	61	8.63			
				Healthy	67	1.06			
		*S. aureus*		Subclinical	12	4.38			
		NAS		Subclinical	14	1.57			
							65	76	1.28
							77	83	1.81
Thomas et al. 2015	*Range*		*Composite*			*Median / Range*			
	9 - 6,154			All	54	1.17 / <0.6-50.13			
	<100			Healthy	29	0.6 / <0.6-50.13			
	101 - 200			Subclinical	8	0.6 / <0.6			
	>200			Clinical	17	0.6 / <0.6-24.81			
Hussein et al. 2018			*Quarter*			*Mean*			
	≤500			SCC group 1	148	3.58			
	≥500			SCC group 2	72	35.2			
Bochniarz et al. 2020			*Quarter*			*Median / Range*			
		*S. uberis*		Clinical	14	2.59 / 0.47-5.84			
		*S. agalactiae*			7	3.88 / 0.88-7.60			
		*Strep.* spp.			30	1.13 / 0.42-7.60			
	<100	Negative		Healthy	10	0.32 / 0.15-0.51			
Jaeger et al. 2017	*Median (IQR)*		*Composite*			*Median (IQR)*			
	813 (158-2,512)	*S. aureus*			26	8.19 (1.52-49.85)			
	309 (68-1,288)	*S. uberis*			14	6.27 (0.28-30.01)			
	295 (16-3,090)	Other major			16	5.19 (0.19-14.78)			
	457 (25-2,818)	Major			56	6.68 (0.16-41.87)			
	178 (19-1,023)	NAS			76	3.24 (0.00-13.71)			
	240 (30-946)	*C. bovis*			109	3.63 (0.00-14.75)			
	257 (21-933)	NAS + *C. bovis*			28	3.60 (0.00-16.19)			
	62 (11-331)	Other minor			6	1.40 (0.42-5.16)			
	234 (18-1,047)	Minor			219	3.44 (0.00-14.68)			
	109 (10-813)	Negative			158	1.28 (0.00-14.75)			
Dalanezi et al. 2020*			*Individual*			*Median (IQR) / Range*			
		*K. pneumoniae*		Clinical	18	52.4 (31.9-97.1) / 2.0-178.8			
		*E. coli*			24	20.5 (8.6-47.2) / 0.0-264.0			
		*S. aureus*			15	38.2 (21.8-94.9) / 13.3-129.5			
		*Strep.* spp.			16	63.8 (48.4-70.6) / 21.0-151.4			
		*Mycoplasma* spp.			18	35.2 (17.4-51.5) / 0.0-102.0			
		*Enterococcus* spp.			18	55.4 (26.8-86.1) / 0.0-250.0			
		NAS			24	14.9 (8.7-37.7) / 0.0-141.7

**Table 3:** Cow, results obtained for haptoglobin by ELISA. The unit of measurement is μg/mL.

Study	SCC (cells/ml x 10^3^)	Pathogens	Sample	Sample group	N	Value	%Se	%Sp	cutoff
Hiss et al. 2007	*Median / Range*		*Quarter*			*Median / Range*			
	40 / 4-1,512	Negative		Group 1	79	0.70 / 0.35-16.0			
	70,500 / 6-2,249	*C. bovis*		Group 2	70	1.85 / 0.35-85.0			
	136 / 12-10,000	Mixed inf.		Group 3	45	2.4 / 0.5-150.2			
	167 / 9-4,171	NAS		Group 4	60	3.1 / 0.35-576.0			
	405 / 32-10,000	*Strep.* spp.		Group 5	29	4.4 / 0.50-974.0			
	335 / 8-8,804	Mixed + *S. aureus*		Group 6	35	4.8 / 0.35-232.4			
	1,741 / 16-10,000	*S. aureus*		Group 7	49	39.6 / 0.35-304.8			
	8,861 / 1,658-10,000	*E. coli*		Group 8	3	81.0 / 59.0-184.0			
							85	92	2.2 (SCC 100)
							89	92	2.7 (SCC 200)
Kováč et al. 2007			*Composite*			*Median / Range*			
	<100			Group 1	7	0 / 0-0			
	101-400			Group 2	7	0 / 0-0.68			
	>400			Group 3	8	6.76 / 0-20.0			
Safi et al. 2009	*Median / Range*		*Quarter*			*Median / Range*			
	1,250 / 3-51,840	*S. aureus, Strep. ag.*		Subclinical	39	10 / 0-1,382			
	75 / 3-266	Negative		Healthy	134	0 / 0-500			
							90.6	68.6	3.9
Zeng et al. 2009			*Quarter*			*Mean (SD)*			
	< 250			SCC group 1	347	0.50 (0.15)			
	> 250			SCC group 2	46	7.18 (2.10)			
Wenz et al. 2010			*Quarter*			*Mean (95% CI)*			
				Clinical (mild)	87	503 (344-735)			
				Clinical (moderate-severe)	60	1,013 (644-1594)			
		Gram-negative			83	1,126 (759-1,670)			
		Gram-positive			64	575 (375-881)			
		NAS			19	403 (196-828)			
		*Strep.* spp.			45	686 (418-1,127)			
		*E. coli*			57	1,052 (675-1,639)			
		Coliforms			26	1,370 (704-2,666)			
Pyörälä et al. 2011			*Quarter*			*Median (IQR)*			
		*S. aureus*			44	33.0 (<7.8-95.3)			
		NAS			45	7.8 (<7.8-73)			
		*Strep. uberis*			43	36.7 (<7.8-249.5)			
		*E. coli*			23	243.5 (32.1-625)			
		*A. pyogenes*			24	440.3 (164.5-961.5)			
		*Strep. dysgalactiae*			48	34.5 (<7.8-125.5)			
		Other bacteria			7	<7.8 (<7.8-159.5)			
				Subclinical	136	33.8 (<7.8-135.5)			
				Clinical	98	80 (10.2-332.0)			
	>200 >100						52.974.0	94.664.4	
Thomas et al. 2015	*Median / Range*					*Median / Range*			
			Quarter	All	149	3.60 / <0.4-420			
	101-200		Composite	Subclinical	8	4.02 / <0.4-5.28			
	>200			Clinical	17	6.40 / <0.4-55.46			
	96 / 9-6,154			All	54	3.46 / <0.4-55.46			
	<100			Healthy	29	2.96 / <0.4-13.74			
Dalanezi et al. 2020			*Individual*			*Median (IQR) / Range*			
		*K. pneumoniae*		Clinical	18	206.1 (126.3-468.1) / 0.0-1,113.3			
		*E. coli*			24	164.1 (71.7-305.1) / 0.0-2,009.4			
		*S. aureus*			15	158.7 (0.0-300.0) / 0.0-596.1			
		*Strep*. spp.			16	179.0 (130.2-363.7) / 0.0-812.2			
		*Mycoplasma* spp.			18	102.0 (0.0-332.8) / 0.0-582.9			
		*Enterococcus* spp.			18	43.0 (0.0-127.3) / 0.0-213.0			
		NAS			24	0.0 (0.0-66.3) / 0.0-319.1			
Wollowski et al. 2021			*Quarter*			*Mean*			
				Subclinical	107	10.15			
				Clinical (all)	115	13.73			
				Healthy	67	0.98			
		*Strep. uberis*		Subclinical	22	11.1			
		*S. aureus*		Subclinical	12	11.86			
		NAS		Subclinical	14	8.52			
							92	94	3.65
							96	99	5.40

**Table 4:** Cow, results obtained for other non-cytokine markers.

Study	SCC (cells/ml x 10^3^)	Pathogens	Marker	Assay	Unit	Sample	Sample group	N	value	%Se	%Sp	Cutoff
Dalanezi et al. 2020			*AGP*	*SPARCL*	*μg/ml*	*Individual*	*Clinical*		*Median (IQR) / Range*			
		*K. pneumoniae*						18	34.9 (22.4-145.3) /14.4-305.0			
		*E. coli*						24	47.3 (24.8-155.7) / 6.8-892.9			
		*S. aureus*						15	51.3 (17.3-207.4) / 11.9-289.9			
		*Streptococcus* spp.						16	29.7 (19.0-49.3) / 11.3-100.7			
		*Mycoplasma* spp.						18	24.7 (15.1-79.2) / 10.6-427.5			
		*Enterococcus* spp.						18	17.9 (15.8-22.2) / 10.1-297.5			
		NAS						24	9.7 (7.7-9.7) / 5.9-42.6			
Wenz et al. 2010			*BSA*	*ELISA*	*mg/mL*	*Quarter*			*Mean*			
							Clinical (mild)	87	4.34			
							Clinical (moderate-severe)	60	5.37			
		Gram-negative						83	6.08			
		Gram-positive						64	3.85			
		NAS						19	3.22			
		*Streptococcus* spp.						45	4.09			
		*E. coli*						57	6.39			
		Coliforms						26	5.38			
Addis et al. 2017	*Median (IQR)*		*CATH*	*ELISA*	*AOD450*	*Quarter*			*Median (IQR):*			
	7.5 (3-21)						Healthy	100	0.089 (0.084-0.094)			
	5,588 (2,540-7,814)						Clinical	435	11.850 (3.090-27.120)			
	6,543 (4,316-8,409)	Mixed infection						25	8.970 (3.023-30.100)			
	6,362 (4,586-7,992)	*S. agalactiae*						59	27.480 (12.290-30.100)			
	5,818 (3,748-8,665)	Other bacteria						36	6.420 (2.454-20.827)			
	5,513 (3,108-7,368)	*S. aureus*						14	16.165 (7.071-22.302)			
	5,522 (2,762-7,521)	Gram-negative						102	11.640 (4.805-22.302)			
	5,405 (2,461-7,394)	*Strep.* spp.						99	13.020 (3.727-30.000)			
	5,049 (1,106-7,992)	Negative						59	10.620 (2.803-18.920)			
	3,037 (1,078-6,146)	NAS						41	3.120 (0.866-9.055)			
										99.08	100	0.115
Wollowski et al. 2021			*CATH*	*ELISA*	*NOD450*	*Quarter*			*Mean*			
							Subclinical	107	0.951			
							Clinical (all)	115	2.420			
							Healthy	67	0.001			
		*Strep. uberis*					Subclinical	22	1.238			
		*S. aureus*					Subclinical	12	1.309			
		NAS					Subclinical	14	0.326			
										83	97	0.00
										98	99	0.053
Dalanezi et al. 2020			*CRP*	*SPARCL*	*μg/ml*	*Individual*			*Median (IQR) / Range*			
		*K. pneumoniae*					Clinical	18	1.6 (0.5-4.4) / 0.1-8.0			
		*E. coli*						24	2.0 (0.8-4.8) / 0.1-7.8			
		*S. aureus*						15	0.8 (0.1-2.0) / 0.0-9.2			
		*Strep.* spp.						16	0.6 (0.3-1.4) / 0.1-5.4			
		*Mycoplasma* spp.						18	0.6 (0.3-2.1) / 0.1-6.8			
		*Enterococcus* spp.						18	0.2 (0.1-0.7) / 0.1-2.6			
		NAS						24	0.1 (0.1-0.1) / 0.1-3.5			
*Galfi et al. 2016*			*IG*	*RID*	*g/L*	*Quarter*			*Mean (SD) / Range*			
							Healthy	76	24.64 (23.56) / 4.78-162.38			
							Subclinical	74	29.35 (24.38) / 4.62-152.24			
		*S. aureus*						3	12.21 (8.08)			
		*Strep. agalactiae*						3	22.51 (15.18)			
		*Strep. dysgalactiae*						2	28.81 (15.88)			
		*E. faecium*						1	23.19			
		*Coryne.* spp.						49	31.15 (27.30)			
		NAS						7	34.11 (21.42)			
		Other bacteria						9	24.68 (17.51)			
Chen et al. 2004			*LF*	*ELISA*	*μg/ml*	*Individual*			*Mean (SD)*			
	<100						SCC group 1	50	176.8 (120.3)			
	100-250						SCC group 2	15	466.0 (508.5)			
	250-500						SCC group 3	10	742.1 (374.2)			
Cheng et al. 2008			*LF*	*ELISA*	*μg/ml*	*Individual*			*Mean (SD)*			
	0-18						SCC group 0	12	log 1.914 (0.137)			
	18-35						SCC group 1	20	log 2.022 (0.174)			
	35-71						SCC group 2	50	log 1.980 (0.191)			
	71-141						SCC group 3	40	log 2.058 (0.264)			
	141-283						SCC group 4	34	log 2.098 (0.245)			
	283-566						SCC group 5	20	log 2.262 (0.317)			
	566-1,131						SCC group 6	22	log 2.276 (0.303)			
Sobczuk-Szul et al. 2014			*LF*	*ELISA*	*μg/ml*	*ND*			*Mean*			
	<100						SCC group 1	56	149.16			
	101-400						SCC group 2	66	212.69			
	400-1,000						SCC group 3	21	233.20			
	>1,000						SCC group 4	18	246.77			
*Galfi et al. 2016*			*LF*	*ELISA*	*mg/ml*	*Quarter*			*Mean (SD) / Range*			
							Healthy	76	5.12 (1.77) / 0.73-8.85			
							Subclinical	74	5.94 (1.65) / 2.26-9.84			
		*S. aureus*						3	6.21 (0.50)			
		*Strep. agalactiae*						3	6.48 (0.51)			
		*Strep. dysgalactiae*						2	4.71 (0.38)			
		*E. faecium*						1	5.88			
		*Coryne.* spp.						49	6.05 (1.68)			
		NAS						7	5.30 (1.36)			
		Other bacteria						9	5.88 (2.35)			
*Galfi et al. 2016*			*LF*	*ELISA*	*mg/ml*	*Quarter*			*Mean (SD) / Range*			
		Major						10	5.45 (1.69) / 1.17-6.88			
		Contagious						7	5.61 (2.00) / 1.17-6.88			
		*S. aureus*						4	4.95 (2.55) / 1.17-6.68			
		*Strep. agalactiae*						3	6.48 (0.50) / 5.9-6.88			
		Environmental						3	5.10 (0.68) / 4.68-5.88			
		*Strep. dysgalactiae*						2	4.71 (0.04) / 4.68-4.73			
		*E. faecium*						1	5.88			
		Minor						56	5.95 (1.65) / 2.40-9.84			
		*Coryne.* spp.						49	6.05 (1.68) / 2.40-9.84			
		NAS						7	5.30 (1.36) / 4.07-7.60			
		Other bacteria						9	5.88 (2.35) / 2.26-8.34			
		Negative					Healthy	76	5.12 (1.77) / 0.73-8.85			
Zeng et al. 2009			*LBP*	*ELISA*	*μg/ml*	*Quarter*			*Mean (SD)*			
	< 250						SCC group 1	347	6.20 (0.33)			
	> 250						SCC group 2	46	12.78 (1.39)			
*Wenzet al. 2010*			*LBP*	*ELISA*	*μg/ml*	*Quarter*			*Mean (95% CI)*			
							Clinical (mild)	87	33.3 (25.1-44.3)			
							Clinical (moderate-severe)	60	60.9 (43.8-84.8)			
		Gram-negative						83	51.0 (38.0-68.5)			
		Gram-positive						64	35.2 (25.6-48.4)			
		NAS						19	42.4 (24.7-73.0)			
		*Strep.* spp.						45	32.6 (22.4-47.3)			
		*E. coli*						57	50.7 (36.3-70.8)			
		Coliforms						26	51.5 (31.2-85.0)

**Table 5:** Cow, results obtained for cytokine markers by ELISA.

Study	SCC (cells/ml x 10^3^)	Pathogens	Marker	Unit	Sample	Sample group	N	Value	%Se	%Sp	Cutoff
Wenz et al. 2010			*IL-10*	*U/mL*	*Quarter*			*Mean (95% CI)*			
						Clinical (mild)	87	16.9 (11.3-25.5)			
						Clinical (moderate-severe)	60	48.9 (30.1-79.5)			
						Gram-negative	83	48.7 (31.9-74.2)			
						Gram-positive	64	16.3 (10.3-25.8)			
		NAS					19	7.60 (3.55-16.3)			
		*Strep.* spp.					45	21.8 (12.9-36.9)			
		*E. coli*					57	53.6 (33.6-85.6)			
		Coliforms					26	39.0 (19.3-78.8)			
Wenz et al. 2010			*IL-12*	*U/mL*	*Quarter*			*Mean (95% CI)*			
						Clinical (mild)	87	153 (102-229)			
						Clinical (moderate-severe)	60	339 (213-539)			
						Gram-negative	83	210 (138-320)			
						Gram-positive	64	270 (172-426)			
		NAS					19	186 (86-402)			
		*Strep.* spp.					45	313 (185-532)			
		*E. coli*					57	218 (136-350)			
		Coliforms					26	195 (96-396)			
Wenz et al. 2010			*IL-1β*	*ng/mL*	*Quarter*			*Mean (95% CI)*			
						Clinical (mild)	87	2.59 (2.00-3.35)			
						Clinical (moderate-severe)	60	4.22 (3.15-5.66)			
		Gram-negative					83	4.35 (3.33-5.68)			
		Gram-positive					64	2.55 (1.91-3.41)			
		NAS					19	1.72 (1.06-2.79)			
		*Strep.* spp.					45	2.99 (2.14-4.17)			
		*E. coli*					57	4.50 (3.34-6.06)			
		Coliforms					26	4.04 (2.58-6.32)			
Sobczuk-Szul et al. 2014			*IL-1β*	*ng/ml*	*ND*			*Mean*			
	<100					SCC group 1	56	0.08			
	101-400					SCC group 2	66	0.15			
	400-1,000					SCC group 3	21	0.19			
	>1,000					SCC group 4	18	0.21			
Sakemi et al. 2011	*Mean / Range*		*IL-6*	*pg/ml*	*Quarter*			*Mean (SD)*			
	36 / 11-74					SCC Low	37	12.6 (33.4)			
	770 / 130-3,310					SCC High	40	207.0 (441.6)			
Sobczuk-Szul et al. 2014			*IL-6*	*ng/ml*	*ND*			*Mean*			
	<100					SCC group 1	56	0.06			
	101-400					SCC group 2	66	0.11			
	400-1,000					SCC group 3	21	0.08			
	>1,000					SCC group 4	18	0.04			
									40	92	80
Wenz et al. 2010			*TGF-α*	*pg/mL*				*Mean*			
					Quarter	Clinical (mild)	87	109			
						Clinical (moderate-severe)	60	168			
		Gram-negative					83	137			
		Gram-positive					64	107			
		NAS					19	91			
		*Strep.* spp.					45	111			
		*E. coli*					57	151			
		Coliforms					26	107			
Wenz et al. 2010			*TGF-β1*	*ng/mL*	*Quarter*			*Mean*			
						Clinical (mild)	87	6.80			
						Clinical (moderate-severe)	60	6.61			
		Gram-negative					83	7.20			
		Gram-positive					64	7.08			
		NAS					19	7.66			
		*Strep.* spp.					45	7.07			
		*E. coli*					57	6.58			
		Coliforms					26	9.11			
Sobczuk-Szul et al. 2014			*TNF-𝜶*	*ng/ml*	*ND*			*Mean*			
	<100					SCC group 1	56	0.81			
	101-400					SCC group 2	66	0.63			
	400-1,000					SCC group 3	21	0.90			
	>1,000					SCC group 4	18	0.08			

***Sheep.*** Out of 4 papers on sheep milk, 2 (40.0%) assessed CATH, while 1 each (20.0%) were on interleukins and M-SAA, respectively. ELISA was used in all studies, three of which were observational (60.0%) and 2 (40.0%) experimental. All studies were carried out on half-udder milk samples. Table 6 summarizes the main findings of the 4 papers.

**Table 6:** Sheep, results obtained for all markers by ELISA.

Study	SCC (cells/ml x 10^3^)	Pathogens	Marker	Unit	Sample	Sample group	N.	Results	%Se	%Sp	Cutoff
Winter et al. 2002*			*Cytokines*	*ng/ml*	*Half-udder*			*Mean (SD)*			
			IL-1β IL-6 IL-8			NA peak at 8h p.i. peak at 8h p.i.	10 10 10	NA 2.1 (2.1) 45.5 (4.1)			
Miglio et al. 2013			*M-SAA*	*μg/mL*	*Half-udder*			*Mean (SD) / Range*			
		NAS, *S. aureus,* *E. faecalis, S. uberis*				Latent mastitis Subclinical mastitis	11 25	44.59 (42.07) / 7.77-137.09 114.37 (41.14) / 18.41-142.43			
*Addis et al. 2016*			*CATH*	*NOD450*	*Half-udder*						
	>500 >1,000					SCC group 1 SCC group 2			86.2 93.4	94.6 94.3	0.014 0.040
*Puggioni et al. 2020*	*Median (IQR)*		*CATH*	*AOD450*	*Half-udder*	*Late lactation*		*Median (IQR)*			
	235 (122.5-554.5) 1,637 (842.8-14,422)	NAS, *E. faecalis,* *S. uberis, Klebsiella* spp.				Culture negative Culture positive	281 34	0.0861 (0.0701-0.1071) 0.2261 (0.1352-2.275)	91.2	82.9	0.121

*Experimental infection study

#### Goat

Two (66.7%) out of 3 studies assessed CATH, while 1 (33.3%) assessed LF. ELISA was used in all studies, which are all observational. All papers investigated biomarkers from half-udder, but one (Chen et al. 2004) used also bulk milk samples. Table 7 summarizes the main findings of the three papers.

**Table 7:** Goat, results obtained for all markers by ELISA.

Study	SCC (cells/ml x 10^3^)	Pathogens	Marker	Unit	Sample	Sample group	N.	Results	%Se	%Sp	Cutoff
Chen et al. 2004			*LF*	*μg/ml*	*bulk*			*Mean (SD)*			
						MBRT (>8hr) MBRT (5-8hr) MBRT (<5hr) MBRT (4-4.5hr) 0-72hr p.i.	54 47 5 10 3	167 (49) 218 (77) 304 (87) 587 (120) Range: 10-30			
Tedde et al. 2019			*CATH*	*NOD450*	*half-udder*						
							360		85.71	40.41	0.014
*Puggioni et al. 2020*	*Median (IQR)*		*CATH*	*AOD450*	*half-udder*	*Late lactation*		*Median (IQR)*			
	303 (104-772.5) 812.5 (232.3-2,397)	NAS, *S. aureus, S. dysgalactiae*				Culture negative Culture positive	189 34	0.1121 (0.0886-0.1501) 0.1148 (0.1152-0.2449)	76.47	57.14	0.118

#### Water buffalo

Only two observational studies were performed on buffalo. The biomarkers investigated were LF and CATH from quarter milk by ELISA. Table 8 summarizes the main findings of the two papers.

**Table 8:** Water buffalo, results obtained for all markers by ELISA.

**Study**	**SCC (cells/ml x 10**^**3**^**)**	**Pathogens**	**Marker**	**Unit**	**Sample**	**Sample group**	**N**	**Value**	**%Se**	**%Sp**	**Cutoff**
*Ozenc et al. 2019*	*Range*		*LF*	*log*	*Quarter*			*Mean (SD)*			
	0-18 18-36 36-71 71-142 0-18 18-36 36-71 71-142 142-283 283-566 566-1,132 1,132-2,263 2,263-4,536					SCC group 0 SCC group 1 SCC group 2 SCC group 3 SCC group 0 SCC group 1 SCC group 2 SCC group 3 SCC group 4 SCC group 5 SCC group 6 SCC group 7 SCC group 8	194 77 63 57 7 7 9 12 17 11 9 7 5	1.22 (0.21) 1.46 (0.30) 1.43 (0.26) 1.51 (0.25) 1.51 (0.33) 1.67 (0.17) 1.73 (0.38) 1.68 (0.36) 1.82 (0.43) 1.91 (0.53) 2.01 (0.55) 2.14 (0.57) 2.58 (0.40)			
		Total *S. aureus* *NAS* *Candida spp.* *S. agalactiae* *Bacillus spp.* *E. coli* *S. aureus/Candida spp.* *S. aureus/*NAS *E. coli/*NAS					84 13 23 26 6 5 4 3 2 2	1.85 (0.47) 2.22 (0.57) 1.74 (0.40) 1.69 (0.38) 2.25 (0.48) 1.77 (0.27) 1.64 (0.48) 2.08 (0.60) 2.20 (0.08) 1.57 (0.18)			
									97.62 91.67 82.14 47.62 29.76	25.83 61.89 78.77 92.07 97.70	15.01 25.4 32.9 50.3 101.7
*Puggioni et al. 2020*	*Median (IQR)*		*CATH*	*AOD450*	*Quarter*			*Median (IQR)*			
	222 (64-985)					Subclinical mastitis	235	0.120 (0.100–0.189)			
	4,091 (2,789-5,173)					Clinical mastitis	7	0.306 (0.133-0.485)			
	324 (103.5–1,556)	*S. aureus*					221	0.112 (0.094–0.138)			
	105 (46–473.5)	NAS					137	0.102 (0.092-0.122)			
	77 (37.2-348)	Other					126	0.110 (0.090-0.131)			
	90 (45-382)	Sterile					63	0.103 (0.090-0.118)			
	259 (59.5-721)	*Strep.* spp.*/Ent.* spp.					49	0.105 (0.080-0.1305)			
	199.5 (62.5-667.3)	*Gram-negatives*					22	0.110 (0.090-0.131)			
									57.85	84.09	

### Biomarker overview

Table 1 summarizes our results presented in descending order of records addressing biomarkers and dairy species. Among all markers, M-SAA was the most frequently mentioned (n. 16; 48.5%), followed by HP (n. 9; 27.3%;), CATH (n. 8; 24.2%) and LF (n. 7; 21.2%;). Other markers investigated were IL1β and IL6, addressed in 3 papers each (9.1%), followed by IgG (n. 2; 6.1%) and finally AGP, BSA, CRP, IL8, IL10, IL12, LBP, TGFα, TGFβ, TNFα (n. 1; 3.0%).

#### Milk serum amyloid (M-SAA)

M-SAA is produced extrahepatically by healthy mammary epithelial cells (McDonald et al. 2001; Larson et al. 2005) and during inflammation (Grönlund et al. 2003; Larson et al. 2005; Brenaut et al. 2014). M-SAA was the protein most investigated as subclinical mastitis marker in ruminant milk, particularly in dairy cows (Table 2). In our study, we observed that in 17 papers M-SAA was investigated predominantly by ELISA with the commercial kit Tridelta solid sandwich ELISA in two variants (Tridelta Mast ID range MAA assay, Tridelta Development Ltd., Kildare, Ireland, Cat. No.: TP-802 for serum and TP-807 for milk). However, to diagnose mastitis, the authors did not discriminate for serum or milk amyloid isoforms but for the different matrices, defining the protein as SAA when analyzing serum and M-SAA when analyzing milk, respectively. Interestingly, in 5 studies M-SAA was investigated only by TP-802 (Grönlund et al. 2005; Eckersall et al. 2006b; Kováč et al. 2007; Åkerstedt et al. 2007, 2009), in 5 only by TP-807 (Åkerstedt et al. 2011; Shirazi-Beheshtiha et al. 2011; Jaeger et al. 2017; Hussein et al. 2018; Bochniarz et al. 2020; Wollowski et al. 2021), in 2 by both TP-802 and TP-807 (Gerardi et al. 2009; Safi et al. 2009) and in 5 a Tridelta kit was used but the test category was unspecified (Suojala et al. 2008; Pyörälä et al. 2011; Kovačević-Filipović et al. 2012; Szczubiał et al. 2012; Thomas et al. 2015). In particular, Gerardi et al. (2009) investigated M-SAA in milk with both TP-807 and TP-802 assays to compare their diagnostic performances. The sensitivity of TP-807 test is 0.10 μg/ml but a cut-off able to discriminate healthy from mastitic milk has not been defined yet. Miglio et al. (2013) reported a M-SAA peak almost 10 times higher in sheep milk than cow milk. Although no official reference range is fixed for M-SAA in milk, healthy sheep milk concentration ranges from 23.75 to 35.61 μg/ml (Miglio et al. 2013), higher than that observed in cow milk (range: 0.0 - 7.5 μg/ml) (Gerardi et al. 2009). In goat, the MAA as mastitis marker was not suitable. In this species, M-SAA levels increase physiologically as lactation progresses as does SCC, even in absence of infection (Pisanu et al. 2020).

#### Haptoglobin (HP)

HP was the second most represented marker in our literature search. Its performance for mastitis detection was analyzed in 9 records, only for cows and by ELISA (Table 3). HP found in milk has an undefined origin. However, similarly to M-SAA, extrahepatic production may also occur in the mammary tissue. Still, it has been demonstrated that HP concentration increases in milk upon endotoxin challenge, experimental, and natural intramammary infection (IMI) (Grönlund et al. 2003; Eckersall et al. 2006; Gerardi et al. 2009). Interestingly, HP appears in milk and raises in level 3 hours and in blood 9 hours after inflammation (Hiss et al. 2004), indicating that the production of this biomarker by the mammary gland is rapid and specific. The diagnostic performance reported in cows by various authors is promising (Table 3) and encourages its evaluation also in other dairy species. For its characteristics, this biomarker might also be promising for the diagnosis of caprine mastitis, particularly in late lactation, when the SCC is high and other markers fail to provide satisfactory performances (Pisanu et al. 2020).

#### Cathelicidin (CATH)

CATH was measured mainly most by ELISA in goats (n. 3), cows (n. 2), sheep (n. 2), and water buffalo (n. 1). CATH are host defense proteins with antimicrobial and immunomodulatory functions (van Harten et al. 2018) produced by milk PMNs (Kościuczuk et al. 2012) and mammary epithelial cells (Zanetti 2004, 2005; Addis et al. 2013; Cubeddu et al. 2017). The ruminant genome contains numerous CATH proteoform genes, but their differential abundance in mastitic milk is poorly known (Zanetti 2005). CATH showed a high diagnostic performance especially in cows and sheep, also in late lactation. Interestingly, by using a threshold set using negative healthy controls, a good sensitivity of the dedicated ELISA is reached not only for cow and sheep milk (Addis et al. 2016a, 2016b), but also for water buffalo milk (Puggioni et al. 2020a). Conversely, the application of CATH-ELISA in goats remains unsatisfactory in late lactation, especially in pluriparous goats. In fact, the related physiological increase in PMN compromises its reliability, as mentioned above for M-SAA (Pisanu et al. 2020).

#### Lactoferrin (LF)

LF was primarily detected by ELISA in studies involving cows (n. 5), goats (n. 1,) and water buffalo (n. 1). LF is a glycoprotein of the immune defense secreted by mammary epithelial cells during the late stage of milking and mammary involution (Welty et al. 1976; Galfi et al. 2016a). The presence of LF in milk is due to secretion by epithelial cells and degranulation of PMNs during inflammation (Lash et al. 1983). Even though LF is not an APP, it increases remarkably during the inflammatory response due to its production by mammary epithelial cells (Galfi et al. 2016a). Concerning test characteristics for goats and cows, two studies carried out a competitive ELISA by using a lactoferrin antiserum from rabbit, and goat lactoferrin was isolated and purified (Chen and Mao 2004; Chen et al. 2004). In other studies, cow LF was quantified by a commercial sandwich LF ELISA kit (Bethyl Laboratories, Montgomery, TX) (Cheng et al. 2008; Sobczuk-Szul et al. 2014; Galfi et al. 2016a, 2016b). For water buffalo, a specific ELISA kit was produced for the study (Özenç et al. 2019). None of the studies reported test characteristics for LF, and therefore no information on sensitivity or specificity is available for this marker.

#### Other markers

IL1β and IL6 were studied in both cows and sheep (Tab.2), IL-8 only in sheep, and the other proteins (AGP, BSA, CRP, IG, IL10, IL12, LBP, TGFα, TGFβ, TNFα) only in cows. In humans, immune cytokines such as TNFα, INFγ, and ILs are investigated as inflammatory markers to detect subclinical mastitis and identify Th1/Th2 ratio in the inflammatory process (Tuaillon et al. 2017). CRP was studied as a predictor of severity of symptomatology in women's breast inflammation (Fetherston et al. 2006).

In cows, immune cells and their related cytokines have been the subject of recent studies (Gulbe et al. 2020; Shaheen et al. 2020), especially pro-inflammatory immune mediators. In other dairy ruminants, however, these proteins and their roles in mastitis have to be still studied and understood.

### Method overview

Clinical signs, SCC or CMT, and bacteriological culture results were the reference standard methods used to define the presence of mastitis or IMI in dairy ruminants, in association or alone (Chakraborty et al. 2019). Among the analytical techniques applied to evaluating protein biomarkers, ELISA was used in 31 of 33 (93.9%) selected records, whereas SPARCL (Spatial Proximity Analyte) and RID (radial immunodiffusion) were each applied in 1 paper.

### Limitations of the systematic review

#### Issues in research methodology

Our research encountered several critical issues in applying the PRISMA standard methodology, especially concerning the search strategy. While selecting the best performing keywords for carrying out our review, we assessed several combinations for finding those enabling to collect the most comprehensive but selective set of publications possible. During the process, we had some unexpected findings; for instance, including the keyword “ruminant” produced a less sensitive search, leading to the decision to remove it. Interestingly, this gives a clue that the word “ruminant” is uncommonly used in title, abstract, or keywords, probably because the authors prefer to report only the name of the dairy species. Furthermore, misleading titles and abstracts led to identifying papers that did not address the research question, and these had to be excluded (as detailed in Methods). On the other hand, we compensated for the possible loss of records consequent to improper index terms with an additional critical revision of the literature performed on PubMed by an expert author. Furthermore, the references of each retrieved article were screened as a further compensative measure. Nevertheless, there is always a risk for exclusion for those articles that do not contain at least one of the selected search terms in the title, abstract, or keywords. Therefore, it is very important that the authors pay particular care when drafting these crucial parts in order to maximize article retrieval.

#### Bias assessment and applicability of studies

Defining quality assessment of primary studies is an essential step in systematic reviews. Therefore, the risk of bias and applicability must be evaluated and scored in all studies, especially those focused on diagnostic accuracy. Hence, we applied QUADAS, a quality assessment tool, to all the selected studies. Concerning the risk of bias (Supplementary Table IX), on animal selection (domain 1) 18/33 (54.5%) studies had a low risk of bias, 15/33 (45.5%) high, and 0/33 (0.0%) unclear risk. Regarding the index test (domain 2), one study out of 33 (3.0%) had a low risk of bias, 29/33 (87.9%) had high risk, and 3/33 (9.1%) unclear risk. For the reference standard (domain 3), we observed a low risk of bias in 22/33 studies (66.7%), high risk in 9/33 (27.3%) and unclear risk in 2/33 (6.1%). Finally, flow and timing (domain 4) showed low risk of bias in 21/33 records (63.6%), high risk in 11/33 (33.3%), and unclear risk in 1/33 (3.0%). Many studies showed low concerns about applicability, especially regarding domain 3 (Supplementary Table X) In detail, in domain 1, low risk was reported in 29/33 (87.9%), high in 4/33 (12.1%), and unclear in 0/53 (0.0%). In domain 2, records had low risk in 25/33 (75.8%), high in 5/33 (15.1%) and unclear 3/33 (9.1%); whereas in domain 3 we observed low risk in 31/33 (93.9%) papers, high in 2/33 (6.1%) and unclear in 0/33 (0.0%).

### Conclusions and recommendations

Our work aimed at analytically assessing the scientific literature describing the use of non-enzymatic milk proteins as mastitis markers in dairy ruminant species with the PRISMA approach. Moreover, we aimed at summarizing and comparing the diagnostic performances of the immunoassays developed for their detection in the milk. As expected, the most frequently mentioned biomarkers were M-SAA, HP, CATH, and LF, which were investigated both in experimental/observational studies and in discovery/implementation approaches. Nonetheless, we observed several critical issues in study designs, reference standard methods (the lack of “gold standard”), index test (frequently performed without a blind approach), heterogeneity in the unit of measurement used for detecting the same biomarker, and the different type of statistical analysis performed, resulting in a heterogeneity of the collected data that was not amenable to meta-analysis. Unfortunately, this is a common finding in many meta-analyses and illustrates how important it is for case definitions and other criteria to be standardized between studies. Nevertheless, being related to the nature of the disease, some of these issues could hardly be solved, even because a truly reliable, sensitive, and specific reference diagnostic test does not exist. To deal with this, we applied an alternative synthesis method newly used in systematic reviews, the *“Synthesis Without Meta-analysis”* (SWiM), which improves transparency in reporting. The critical issues we observed further highlight the importance of title writing and keyword definition, both in the publishing and searching phases. When drafting these crucial parts of their manuscripts, using appropriate consensus terminology will maximize retrieval in bibliographic searches, enhancing article visibility and data usability.

## Supplementary Information

ESM 1 (DOCX 82 kb)

## References

[CR1] Addis MF, Pisanu S, Marogna G, Cubeddu T, Pagnozzi D, Cacciotto C, Campesi F, Schianchi G, Rocca S, Uzzau S (2013). Production and Release of Antimicrobial and Immune Defense Proteins by Mammary Epithelial Cells following Streptococcus uberis Infection of Sheep. Infect Immun.

[CR2] Addis MF, Tedde V, Dore S, Pisanu S, Puggioni GMG, Roggio AM, Pagnozzi D, Lollai S, Cannas A, Uzzau S (2016). Evaluation of milk cathelicidin for detection of dairy sheep mastitis. J Dairy Sci.

[CR3] Addis MF, Tedde V, Puggioni GMG, Pisanu S, Casula A, Locatelli C, Rota N, Bronzo V, Moroni P, Uzzau S (2016). Evaluation of milk cathelicidin for detection of bovine mastitis. J Dairy Sci.

[CR4] Addis MF, Bronzo V, Puggioni GMG, Cacciotto C, Tedde V, Pagnozzi D, Locatelli C, Casula A, Curone G, Uzzau S, Moroni P (2017). Relationship between milk cathelicidin abundance and microbiologic culture in clinical mastitis. J Dairy Sci.

[CR5] Åkerstedt M, Waller KP, Sternesjö Å (2007). Haptoglobin and serum amyloid A in relation to the somatic cell count in quarter, cow composite and bulk tank milk samples. J Dairy Res.

[CR6] Åkerstedt M, Waller KP, Sternesjö Å (2009). Haptoglobin and serum amyloid A in bulk tank milk in relation to raw milk quality. J Dairy Res.

[CR7] Åkerstedt M, Forsbäck L, Larsen T, Svennersten-Sjaunja K (2011). Natural variation in biomarkers indicating mastitis in healthy cows. J Dairy Res.

[CR8] Bergonier D, de Crémoux R, Rupp R, Lagriffoul G, Berthelot X (2003). Mastitis in Dairy Small Ruminants. Vet Res.

[CR9] Bochniarz M, Szczubiał M, Brodzki P, Krakowski L, Dąbrowski R (2020). Serum amyloid A as a marker of cow֨ s mastitis caused by Streptococcus sp. Comp Immunol Microbiol Infect Dis.

[CR10] Brenaut P, Lefèvre L, Rau A, Laloë D, Pisoni G, Moroni P, Bevilacqua C, Martin P (2014). Contribution of mammary epithelial cells to the immune response during early stages of a bacterial infection to Staphylococcus aureus. Vet Res.

[CR11] Campbell M, McKenzie JE, Sowden A, Katikireddi SV, Brennan SE, Ellis S, Hartmann-Boyce J, Ryan R, Shepperd S, Thomas J, Welch V (2020). Thomson H (2020) Synthesis without meta-analysis (SWiM) in systematic reviews: reporting guideline. BMJ.

[CR12] Chakraborty S, Dhama K, Tiwari R, Iqbal Yatoo M, Khurana SK, Khandia R, Munjal A, Munuswamy P, Kumar MA, Singh M, Singh R, Gupta VK (2019). Chaicumpa W (2019) Technological interventions and advances in the diagnosis of intramammary infections in animals with emphasis on bovine population—a review. Vet Q.

[CR13] Chen PW, Chen WC, Mao FC (2004). Increase of lactoferrin concentration in mastitic goat milk. J Vet Med Sci.

[CR14] Chen PW, Mao FC (2004). Detection of lactoferrin in bovine and goat milk by enzyme-linked immunosorbent assay. J Food Drug Anal.

[CR15] Cheng JB, Wang JQ, Bu DP, Liu GL, Zhang CG, Wei HY, Zhou LY, Wang JZ (2008). Factors affecting the lactoferrin concentration in bovine milk. J Dairy Sci.

[CR16] Contreras A, Sierra D, Sánchez A, Corrales JC, Marco JC, Paape MJ, Gonzalo C (2007). Mastitis in small ruminants. Small Rum Res.

[CR17] Cubeddu T, Cacciotto C, Pisanu S, Tedde V, Alberti A, Pittau M, Dore S, Cannas A, Uzzau S, Rocca S, Addis MF (2017). Cathelicidin production and release by mammary epithelial cells during infectious mastitis. Vet Immunol Immunopathol.

[CR18] Dalanezi FM, Schmidt EMS, Joaquim SF, Guimarães FF, Guerra ST, Lopes BC, Cerri RLA, Chadwick C, Langoni H (2020). Concentrations of Acute-Phase Proteins in Milk from Cows with Clinical Mastitis Caused by Different Pathogens. Pathogens.

[CR19] Eckersall PD, Young FJ, Nolan AM, Knight CH, McComb C, Waterston MM, Hogarth CJ, Scott EM, Fitzpatrick JL (2006). Acute phase proteins in bovine milk in an experimental model of Staphylococcus aureus subclinical mastitis. J Dairy Sci.

[CR20] Ezzat Alnakip M, Quintela-Baluja M, Böhme K, Fernández-No I, Caamaño-Antelo S, Calo-Mata P, Barros-Velázquez J (2014). The immunology of mammary gland of dairy ruminants between healthy and inflammatory conditions. J Vet Med.

[CR21] Fetherston CM, Wells JI, Hartmann PE (2006). Severity of mastitis symptoms as a predictor of C-reactive protein in milk and blood during lactation. Breastfeeding Med.

[CR22] Galfi AL, Radinović MZ, Boboš SF, Pajić MJ, Savić SS, Milanov DS (2016). Lactoferrin concentrations in bovine milk during involution of the mammary glands, with different bacteriological findings. Vet Arhiv.

[CR23] Galfi AL, Radinović MZ, Milanov DS, Savić SS, Boboš SF, Pajić MJ (2016). Lactoferrin and immunoglobulin G concentration in bovine milk from cows with subclinical mastitis during the late lactation period. Acta Sci Vet.

[CR24] Gerardi G, Bernardini D, Elia CA, Ferrari V, Iob L, Segato S (2009). Use of serum amyloid A and milk amyloid A in the diagnosis of subclinical mastitis in dairy cows. J Dairy Res.

[CR25] Grönlund U, Hultén C, Eckersall PD, Hogarth C, Persson Waller K (2003). Haptoglobin and serum amyloid A in milk and serum during acute and chronic experimentally induced Staphylococcus aureus mastitis. J Dairy Res.

[CR26] Grönlund U, Hallén Sandgren C, Persson Waller K (2005). Haptoglobin and serum amyloid A in milk from dairy cows with chronic sub-clinical mastitis. Vet Res.

[CR27] Gulbe G, Pilmane M, Saulīte V, Doniņa S, Jermolajevs J, Peškova L, Valdovskaet A (2020). Cells and cytokines in milk of subclinically infected bovine mammary glands after the use of immunomodulatory composition GLP 810. Mediators Inflamm.

[CR28] Hiss S, Mielenz M, Bruckmaier RM, Sauerwein H (2004). Haptoglobin concentrations in blood and milk after endotoxin challenge and quantification of mammary Hp mRNA expression. J Dairy Sci.

[CR29] Hiss S, Mueller U, Neu-Zahren A, Sauerwein H (2007). Haptoglobin and lactate dehydrogenase measurements in milk for the identification of subclinically diseased udder quarters. Veterinarni Medicina.

[CR30] Hughes K, Watson CJ (2018). The mammary microenvironment in mastitis in humans, dairy ruminants, rabbits and rodents: a one health focus. J Mammary Gland Biol Neoplasia.

[CR31] Hussein HA, El-Razik KAEA, Gomaa AM, Elbayoumy MK, Abdelrahman KA, Hosein HI (2018). Milk amyloid A as a biomarker for diagnosis of subclinical mastitis in cattle. Vet World.

[CR32] Iliev PT, Georgieva TM (2019). Acute phase biomarkers of diseases in small ruminants: an overview. Bulg J Vet Med.

[CR33] Jaeger S, Virchow F, Torgerson PR, Bischoff M, Biner B, Hartnack S, Rüegg SR (2017). Test characteristics of milk amyloid A ELISA, somatic cell count, and bacteriological culture for detection of intramammary pathogens that cause subclinical mastitis. J Dairy Sci.

[CR34] Kelly AL, Leitner G, Merin U (2018) Milk Quality and Udder Health: Test Methods and Standards. Reference Module in Food Science, Elsevier, 2018, ISBN 9780081005965, doi: 10.1016/B978-0-08-100596-5.00951-3.

[CR35] Kościuczuk EM, Lisowski P, Jarczak J, Strzałkowska N, Jóźwik A, Horbańczuk J, Krzyżewski J, Zwierzchowski L, Bagnicka E (2012). Cathelicidins: family of antimicrobial peptides. A review. Mol Biol Rep.

[CR36] Kováč G, Popelková M, Tkáčiková Ľ, Burdová O, Ihnát O (2007). Interrelationship between somatic cell count and acute phase proteins in serum and milk of dairy cows. Acta Vet Brno.

[CR37] Kovačević-Filipović M, Ilić V, Vujčić Z, Dojnov B, Stevanov-Pavlović M, Mijačević Z, Božić T (2012). Serum amyloid A isoforms in serum and milk from cows with Staphylococcus aureus subclinical mastitis. Vet Immunol Immunopathol.

[CR38] Lam TJGM, Olde Riekerink RGM, Sampimon OC, Smith H (2009). Mastitis diagnostics and performance monitoring: a practical approach. Ir Vet J.

[CR39] Larson MA, Weber A, Weber AT, McDonald TL (2005). Differential expression and secretion of bovine serum amyloid A3 (SAA3) by mammary epithelial cells stimulated with prolactin or lipopolysaccharide. Vet Immunol Immunopathol.

[CR40] Lash JA, Coates TD, Lafuze J, Baehner RL, Boxer LA (1983). Plasma lactoferrin reflects granulocyte activation in vivo. Blood.

[CR41] Leitner G, Shoshani E, Krifucks O, Chaffer M, Saran A (2000). Milk leucocyte population patterns in bovine udder infection of different aetiology. J Vet Med B Infect Dis Vet Public Health.

[CR42] Lopez-Benavides MG, Dohoo I, Scholl D, Middleton J, Perez R (2012) Interpreting Bacteriological Culture Results to Diagnose Bovine Intramammary Infections. National Mastitis Council Research Committee Report

[CR43] McDonald TL, Larson MA, Mack DR, Weber A (2001). Elevated extrahepatic expression and secretion of mammary-associated serum amyloid A 3 (M-SAA3) into colostrum. Vet Immunol Immunopathol.

[CR44] Menzies PI, Ramanoon SZ (2001). Mastitis of Sheep and Goats. Vet Clin North Am Food Anim Pract.

[CR45] Miglio A, Moscati L, Fruganti G, Pela M, Scoccia E, Valiani A, Maresca C (2013). Use of milk amyloid A in the diagnosis of subclinical mastitis in dairy ewes. J Dairy Res.

[CR46] Moher D, Liberati A, Tetzlaff J, Altman DG (2009). Preferred Reporting Items for Systematic Reviews and Meta-Analyses: The PRISMA Statement. PLoS Med.

[CR47] Oliver SP, Gonzales RN, Hogan JS, Jayarao BM, Owens WE, Owens W (2004). Microbiological procedures for the diagnosis of bovine udder infection and determination of milk quality. 4^th^ Edition. National Mastitis Council, Inc. Verona (WI), USA.

[CR48] Özenç E, Şeker E, Acar D, Koca HB, Yazıcı E, Çelik H, Doğan N, Avcı G, Yılmaz O, Küçükkebapçı M, Uçar M, Baştan A (2019). Milk lactoferrin concentrations in anatolian buffaloes with and without subclinical mastitis. Buffalo Bull.

[CR49] Pedersen LH, Aalbaek B, Røntved CM, Ingvartsen KL, Sorensen NS, Heegaard PM, Jensen HE (2003). Early pathogenesis and inflammatory response in experimental bovine mastitis due to Streptococcus uberis. J Comp Pathol.

[CR50] Persson Y, Olofsson I (2011). Direct and indirect measurement of somatic cell count as indicator of intramammary infection in dairy goats. Acta Vet Scand.

[CR51] Pisanu S, Cacciotto C, Pagnozzi D, Uzzau S, Pollera C, Penati M, Bronzo V, Addis MF (2020). Impact of Staphylococcus aureus infection on the late lactation goat milk proteome: new perspectives for monitoring and understanding mastitis in dairy goats. J Proteomics.

[CR52] Puggioni GMG, Tedde V, Uzzau S, Guccione J, Ciaramella P, Pollera C, Moroni P, Bronzo V, Addis MF (2020). Evaluation of a bovine cathelicidin ELISA for detecting mastitis in the dairy buffalo: comparison with milk somatic cell count and bacteriological culture. Res Vet Sci.

[CR53] Puggioni GMG, Tedde V, Uzzau S, Dore S, Liciardi M, Cannas EA, Pollera C, Moroni P, Bronzo V, Addis MF (2020). Relationship of late lactation milk somatic cell count and cathelicidin with intramammary infection in small ruminants. Pathogens.

[CR54] Pyörälä S, Hovinen M, Simojoki H, Fitzpatrick J, Eckersall PD, Orro T (2011). Acute phase proteins in milk in naturally acquired bovine mastitis caused by different pathogens. Vet Rec.

[CR55] Rossi RS, Amarante AF, Correia LBN, Guerra ST, Nobrega DB, Latosinski GS, Rossi BF, Rall VLM, Pantoja JCF (2018). Diagnostic accuracy of Somaticell, California Mastitis Test, and microbiological examination of composite milk to detect Streptococcus agalactiae intramammary infections. J Dairy Sci.

[CR56] Ruegg PL (2017). A 100-Year Review: Mastitis detection, management, and prevention. J Dairy Sci.

[CR57] Safi S, Khoshvaghti A, Jafarzadeh SR, Bolourchi M, Nowrouzian I (2009). Acute phase proteins in the diagnosis of bovine subclinical mastitis. Vet Clin Pathol.

[CR58] Sakemi Y, Tamura Y, Hagiwara K (2011). Interleukin-6 in quarter milk as a further prediction marker for bovine subclinical mastitis. J Dairy Res.

[CR59] Sargeant JM, O’Connor AM (2020). Scoping Reviews, Systematic Reviews, and Meta-Analysis: Applications in Veterinary Medicine. Front Vet Sci.

[CR60] Shaheen T, Bilal Ahmad S, Rehman MU, Muzamil S, Bhat RR, Hussain I, Bashir N, Mir MUR, Paray BA, Dawood MAO (2020). Investigations on cytokines and proteins in lactating cows with and without naturally occurring mastitis. Journal of King Saud University - Science.

[CR61] Shimazaki K, Kawai K (2017). Advances in lactoferrin research concerning bovine mastitis. Biochem Cell Biol.

[CR62] Shirazi-Beheshtiha SH, Safi S, Rabbani V, Bolourchi M, Ameri M, Khansari MR (2011). The diagnostic value of determination of positive and negative acute phase proteins in milk from dairy cows with subclinical mastitis. Comp Clin Pathol.

[CR63] Smolenski GA, Wieliczko RJ, Pryor SM (2011). The abundance of milk cathelicidin proteins during bovine mastitis. Vet Immunol Immunopathol.

[CR64] Sobczuk-Szul M, Wielgosz-Groth Z, Nogalski Z, Pogorzelska-Przybyłek P (2014) Changes in the content of whey proteins during lactation in cow’s milk with a different somatic cells count. Vet Med Zoot 65(87)

[CR65] Sordillo LM, Streicher KL (2002). Mammary gland immunity and mastitis susceptibility. J Mammary Gland Biol Neoplasia.

[CR66] Souza FN, Blagitz MG, Penna CFAM, Della Libera AMMP, Heinemann MB, Cerqueira MMOP (2012). Somatic cell count in small ruminants: Friend or foe?. Small Rum Res.

[CR67] Suojala L, Orro T, Järvinen H, Saatsi J, Pyörälä S (2008) Acute phase response in two consecutive experimentally induced E. coli intramammary infections in dairy cows. Acta Vet Scand 50(18). 10.1186/1751-0147-50-18

[CR68] Szczubiał M, Dąbrowski R, Kankofer M, Bochniarz M, Komar M (2012). Concentration of serum amyloid A and ceruloplasmin activity in milk from cows with subclinical mastitis caused by different pathogens. Pol J Vet Sci.

[CR69] Tedde V, Bronzo V, Puggioni GMG, Pollera C, Casula A, Curone G, Moroni P, Uzzau S, Addis MF (2019). Milk cathelicidin and somatic cell counts in dairy goats along the course of lactation. J Dairy Res.

[CR70] Thomas FC, Waterston M, Hastie P, Parkin T, Haining H, Eckersall PD (2015). The major acute phase proteins of bovine milk in a commercial dairy herd. BMC Vet Res.

[CR71] Tuaillon E, Viljoen J, Dujols P, Cambonie G, Rubbo PA, Nagot N, Bland RM, Badiou S, Newell ML, Van de Perre P (2017). Subclinical mastitis occurs frequently in association with dramatic changes in inflammatory/anti-inflammatory breast milk components. Pediatr Res.

[CR72] van Harten RM, van Woudenbergh E, van Dijk A, Haagsman HP (2018). Cathelicidins: Immunomodulatory Antimicrobials. Vaccine (Basel).

[CR73] Viguier C, Arora S, Gilmartin N, Welbeck K, O'Kennedy R (2009). Mastitis detection: current trends and future perspectives. Trends in Biotechnol.

[CR74] Welty FK, Larry Smith K, Schanbacher FL (1976). Lactoferrin Concentration During Involution of the Bovine Mammary Gland. J Dairy Sci.

[CR75] Wenz JR, Fox LK, Muller FJ, Rinaldi M, Zeng R, Bannerman DD (2010). Factors associated with concentrations of select cytokine and acute phase proteins in dairy cows with naturally occurring clinical mastitis. J Dairy Sci.

[CR76] Winter P, Colditz IG (2002). Immunological responses of the lactating ovine udder following experimental challenge with Staphylococcus epidermidis. Vet Immunol Immunopathol.

[CR77] Wollowski L, Heuwieser W, Kossatz A, Addis MF, Puggioni GMG, Meriaux L, Bertulat S (2021). The value of the biomarkers cathelicidin, milk amyloid A, and haptoglobin to diagnose and classify clinical and subclinical mastitis. J Dairy Sci.

[CR78] Zanetti M (2004). Cathelicidins, multifunctional peptides of the innate immunity. J Leukocyte Biol.

[CR79] Zanetti M (2005). The role of cathelicidins in the innate host defenses of mammals. Curr Issues Mol Biol.

[CR80] Zeng R, Bequette BJ, Vinyard BT, Bannerman DD (2009). Determination of milk and blood concentrations of lipopolysaccharide-binding protein in cows with naturally acquired subclinical and clinical mastitis. J Dairy Sci.

