# Supplementary material for: Milk proteins as mastitis markers in dairy ruminants - a systematic review
Source: Vet Res Commun. 2022 Feb 23;46(2):329–51. doi: 10.1007/s11259-022-09901-y (PMC9165246; doi:10.1007/s11259-022-09901-y)
Supplement: Supplementary file 1 — (DOCX 82 kb) [file 11259_2022_9901_MOESM1_ESM.docx]

**Supplementary tables**

**Table I.** Organization of the keywords by category and combinations used for the database searches

|  | **Object**  **(OR)** | **Matrix** | **Diagnosis**  **(OR)** | **Assay**  **(OR)** |
| --- | --- | --- | --- | --- |
| **KW** | *- biomarker*  *- marker*  *- amyloid*  *- haptoglobin*  *- cathelicidin*  *- lactoferrin* | *milk* | *- “intramammary infection”*  *- Mastitis* | *- immunoassay*  *- ELISA*  *- “lateral flow”*  *- immunochromatography* |
|  | Marker | milk | mastitis | immunoassay |
|  | Biomarker | milk | mastitis | immunoassay |
|  | Amyloid | milk | mastitis | immunoassay |
|  | Haptoglobin | milk | mastitis | immunoassay |
|  | Cathelicidin | milk | mastitis | immunoassay |
|  | Lactoferrin | milk | mastitis | immunoassay |
|  | Marker | milk | mastitis | ELISA |
|  | Biomarker | milk | mastitis | ELISA |
|  | Amyloid | milk | mastitis | ELISA |
|  | Haptoglobin | milk | mastitis | ELISA |
|  | Cathelicidin | milk | mastitis | ELISA |
|  | Lactoferrin | milk | mastitis | ELISA |
|  | Marker | milk | mastitis | “lateral flow” |
|  | Biomarker | milk | mastitis | “lateral flow” |
|  | Amyloid | milk | mastitis | “lateral flow” |
|  | Haptoglobin | milk | mastitis | “lateral flow” |
|  | Cathelicidin | milk | mastitis | “lateral flow” |
|  | Lactoferrin | milk | mastitis | “lateral flow” |
|  | Marker | milk | mastitis | immunochromatography |
|  | Biomarker | milk | mastitis | immunochromatography |
|  | Amyloid | milk | mastitis | immunochromatography |
|  | Haptoglobin | milk | mastitis | immunochromatography |
|  | Cathelicidin | milk | mastitis | immunochromatography |
|  | Lactoferrin | milk | mastitis | immunochromatography |
|  | Marker | milk | “intramammary infection” | immunoassay |
|  | Biomarker | milk | “intramammary infection” | immunoassay |
|  | Amyloid | milk | “intramammary infection” | immunoassay |
|  | Haptoglobin | milk | “intramammary infection” | immunoassay |
|  | Cathelicidin | milk | “intramammary infection” | immunoassay |
|  | Lactoferrin | milk | “intramammary infection” | immunoassay |
|  | Marker | milk | “intramammary infection” | ELISA |
|  | Biomarker | milk | “intramammary infection” | ELISA |
|  | Amyloid | milk | “intramammary infection” | ELISA |
|  | Haptoglobin | milk | “intramammary infection” | ELISA |
|  | Cathelicidin | milk | “intramammary infection” | ELISA |
|  | Lactoferrin | milk | “intramammary infection” | ELISA |
|  | Marker | milk | “intramammary infection” | “lateral flow” |
|  | Biomarker | milk | “intramammary infection” | “lateral flow” |
|  | Amyloid | milk | “intramammary infection” | “lateral flow” |
|  | Haptoglobin | milk | “intramammary infection” | “lateral flow” |
|  | Cathelicidin | milk | “intramammary infection” | “lateral flow” |
|  | Lactoferrin | milk | “intramammary infection” | “lateral flow” |

**Table II.** MedLine search results

| **Title** | **Authors/Year** | **DOI** |
| --- | --- | --- |
| [C-reactive protein as a new parameter of mastitis] | Schrödl W et al 1995 |  |
| 15-F2t-Isoprostane Concentrations and Oxidant Status in Lactating Dairy Cattle with Acute Coliform Mastitis | Mavangira V et al 2016 | 10.1111/jvim.13793 |
| A pilot study of acute phase proteins as indicators of bovine mastitis caused by different pathogens | Thomas FC et al. 2018 | 10.1016/j.rvsc.2018.06.015 |
| A proteomic perspective on the changes in milk proteins due to high somatic cell count | Zhang L et al. 2015 | 10.3168/jds.2014-9279 |
| A proteomics-based identification of putative biomarkers for disease in bovine milk | van Altena SE et al. 2016 | 10.1016/j.vetimm.2016.04.005 |
| Advances in BHV1(IBR) research | Straub OC. 2001 |  |
| Association of polymorphism within LTF gene promoter with lactoferrin concentration in milk of Holstein cows | Zabolewicz T et al. 2014 | 10.2478/pjvs-2014-0094 |
| Biosensor assay for determination of haptoglobin in bovine milk | Åkerstedt M et al. 2006 | 10.1017/S0022029906001774 |
| Bovine intra-mammary challenge with Streptococcus dysgalactiae spp. Dysgalactiae to explore the effect on the response of Complement activity | Maye S et al. 2017 | 10.1017/S0022029917000292 |
| Cathelicidin production and release by mammary epithelial cells during infectious mastitis | Cubeddu T et al. 2017 | 10.1016/j.vetimm.2017.06.002 |
| Concentration of serum amyloid A and ceruloplasmin activity in milk from cows with subclinical mastitis caused by different pathogens | Szczubiał M et al. 2012 | 10.2478/v10181-011-0149-x |
| Determination of milk and blood concentrations of lipopolysaccharide-binding protein in cows with naturally acquired subclinical and clinical mastitis | Zeng R et al. 2009 | 10.3168/jds.2008-1636 |
| Development of an immunosensor assay for detection of haptoglobin in mastitic milk | Tan X et al. 2012 | 10.1111/j.1939-165X.2012.00468.x |
| Dynamics of experimentally induced Staphylococcus epidermidis mastitis in East Friesian milk ewes | Winter P et al. 2003 | 10.1017/s002202990300606x |
| Early pathogenesis and inflammatory response in experimental bovine mastitis due to Streptococcus uberis | Pedersen LH et al. 2003 | 10.1053/jcpa.2002.0620 |
| Early post parturient changes in milk acute phase proteins | Thomas FC et al. 2016 | 10.1017/S0022029916000297 |
| Effect of intramammary infusion of tumour necrosis factor-alpha on milk protein composition and induction of acute-phase protein in the lactating cow | Watanabe A et al. 2000 | 10.1046/j.1439-0450.2000.00400.x |
| Effects of induced energy deficiency on lactoferrin concentration in milk and the lactoferrin reaction of primary bovine mammary epithelial cells in vitro | Danowski K et al. 2013 | 10.1111/j.1439-0396.2012.01305.x |
| Effects of lactoferrin and milk on adherence of Streptococcus uberis to bovine mammary epithelial cells | Fang W et al. 2000 | 10.2460/ajvr.2000.61.275 |
| Elevated milk soluble CD14 in bovine mammary glands challenged with Escherichia coli lipopolysaccharide | Lee JW et al. 2003 | 10.3168/jds.S0022-0302(03)73832-6 |
| Escherichia coli and Staphylococcus aureus elicit differential innate immune responses following intramammary infection | Bannerman DD et al. 2004 | 10.1128/CDLI.11.3.463-472.2004 |
| Evaluation of a bovine cathelicidin ELISA for detecting mastitis in the dairy buffalo: Comparison with milk somatic cell count and bacteriological culture | Puggioni GMG et al. 2020 | 10.1016/j.rvsc.2019.11.009 |
| Evaluation of milk cathelicidin for detection of bovine mastitis | Addis MF et al. 2016 | 10.3168/jds.2016-11407 |
| Evaluation of milk cathelicidin for detection of dairy sheep mastitis | Addis MF et al. 2016 | 10.3168/jds.2015-10293 |
| Expression of cathelicidins mRNA in the goat mammary gland and effect of the intramammary infusion of lipopolysaccharide on milk cathelicidin-2 concentration | Zhang GW et al. 2014 | 10.1016/j.vetmic.2014.01.029 |
| Expression of the peptidoglycan recognition protein, PGRP, in the lactating mammary gland | Kappeler SR et al. 2004 | 10.3168/jds.S0022-0302(04)73392-5 |
| Facile construction of a molecularly imprinted polymer-based electrochemical sensor for the detection of milk amyloid A | Zhang Z et al. 2020 | 10.1007/s00604-020-04619-7 |
| Factors affecting the lactoferrin concentration in bovine milk | Cheng JB et al. 2008 | 10.3168/jds.2007-0689 |
| Factors associated with concentrations of select cytokine and acute phase proteins in dairy cows with naturally occurring clinical mastitis | Wenz JR et al. 2010 | 10.3168/jds.2009-2819 |
| Generation of an anti-NAGase single chain antibody and its application in a biosensor-based assay for the detection of NAGase in milk | Welbeck K et al, 2011 | 10.1016/j.jim.2010.09.019 |
| Genetic variability of lactoferrin content estimated by mid-infrared spectrometry in bovine milk | Soyeurt H et al. 2007 | 10.3168/jds.2006-827 |
| Gold Nanoparticle Size-Dependent Enhanced Chemiluminescence for Ultra-Sensitive Haptoglobin Biomarker Detection | Nirala NR et al. 2019 | 10.3390/biom9080372 |
| Haptoglobin concentrations in blood and milk after endotoxin challenge and quantification of mammary Hp mRNA expression | Hiss S et al. 2004 | 10.3168/jds.S0022-0302(04)73516-X |
| Identification of lactoferrin-binding proteins in bovine mastitis-causing Streptococcus uberis | Fang W et al. 1999 | 10.1111/j.1574-6968.1999.tb13647.x |
| Immune-associated traits measured in milk of Holstein-Friesian cows as proxies for blood serum measurements | Denholm SJ et al. 2018 | 10.3168/jds.2018-14825 |
| Immunosensing system for rapid multiplex detection of mastitis-causing pathogens in milk | Juronen D et al. 2018 | 10.1016/j.talanta.2017.10.043 |
| Increase in milk metalloproteinase activity and vascular permeability in bovine endotoxin-induced and naturally occurring Escherichia coli mastitis | Raulo SM et al. 2002 | 10.1016/s0165-2427(01)00423-8 |
| Increase of lactoferrin concentration in mastitic goat milk | Chen PW et al. 2004 | 10.1292/jvms.66.345 |
| Increased Epstein-Barr virus in breast milk occurs with subclinical mastitis and HIV shedding | Sanosyan A et al. 2016 | 10.1097/MD.0000000000004005 |
| Influence of bacterial factors on proliferation of bovine mammary epithelial cells | Calvinho LF et al. 2001 |  |
| Innate immune response in experimentally induced bovine intramammary infection with Staphylococcus simulans and S. epidermidis | Simojoki H et al. 2011 | 10.1186/1297-9716-42-49 |
| Interleukin-6 in quarter milk as a further prediction marker for bovine subclinical mastitis | Sakemi Yet al. 2011 | 10.1017/S0022029910000828 |
| Kinetics of cells and cytokines during immune-mediated inflammation in the mammary gland of cows systemically immunized with Staphylococcus aureus alpha-toxin | Riollet C et al. 2000 | 10.1007/s000110050621 |
| Kinetics of local and systemic isoforms of serum amyloid A in bovine mastitic milk | Jacobsen S et al. 2005 | 10.1016/j.vetimm.2004.09.031 |
| Lactoferrin concentrations in bovine milk prior to dry-off | Newman KA et al. 2009 | 10.1017/S0022029909990033 |
| Low-level laser therapy attenuates LPS-induced rats mastitis by inhibiting polymorphonuclear neutrophil adhesion | Wang Y et al. 2014 | 10.1292/jvms.14-0061 |
| Mastitis detection: current trends and future perspectives | Viguier C et al. 2009 | 10.1016/j.tibtech.2009.05.004 |
| Mastitis is associated with IL-6 levels and milk fat globule size in breast milk | Mizuno K et al. 2012 | 10.1177/0890334412455946 |
| Mid-infrared prediction of lactoferrin content in bovine milk: potential indicator of mastitis | Soyeurt H et al. 2012 | 10.1017/S1751731112000791 |
| Milk cathelicidin and somatic cell counts in dairy goats along the course of lactation | Tedde V et al. 2019 | 10.1017/S0022029919000335 |
| Milk cytokines and subclinical breast inflammation in Tanzanian women: effects of dietary red palm oil or sunflower oil supplementation | Filteau SM et al. 1999 | 10.1046/j.1365-2567.1999.00834.x |
| Milk haptoglobin detection based on enhanced chemiluminescence of gold nanoparticles | Nirala NR et al. 2019 | 10.1016/j.talanta.2019.01.027 |
| Milk prostaglandins and electrical conductivity in bovine mastitis | Atroshi F et al. 1987 | 10.1007/BF00361322 |
| Proteomic analysis of the temporal expression of bovine milk proteins during coliform mastitis and label-free relative quantification | Boehmer JL et al. 2010 | 10.3168/jds.2009-2526 |
| Proteomics and pathway analyses of the milk fat globule in sheep naturally infected by Mycoplasma agalactiae provide indications of the in vivo response of the mammary epithelium to bacterial infection | Addis MF et al. 2011 | 10.1128/IAI.00040-11 |
| Relationship between milk cathelicidin abundance and microbiologic culture in clinical mastitis | Addis MF et al. 2017 | 10.3168/jds.2016-12110 |
| Relationship between milk lactoferrin and etiological agent in the mastitic bovine mammary gland | Chaneton L et al. 2008 | 10.3168/jds.2007-0732 |
| Relationship of Late Lactation Milk Somatic Cell Count and Cathelicidin with Intramammary Infection in Small Ruminants | Puggioni GMG et al. 2020 | 10.3390/pathogens9010037 |
| Serum amyloid A isoforms in serum and milk from cows with Staphylococcus aureus subclinical mastitis | Kovačević-Filipović M et al. 2003 | 10.1016/j.vetimm.2011.10.015 |
| Serum C-reactive protein in dairy herds | Lee WC et al. 2003 |  |
| Serum concentration and mRNA expression in milk somatic cells of toll-like receptor 2, toll-like receptor 4, and cytokines in dairy cows following intramammary inoculation with Escherichia coli | Ma JL et al. 2011 | 10.3168/jds.2011-4167 |
| Susceptibility of sows to experimentally induced Escherichia coli mastitis | Ross RF et al. 1983 |  |
| Test characteristics of milk amyloid A ELISA, somatic cell count, and bacteriological culture for detection of intramammary pathogens that cause subclinical mastitis | Jaeger S et al. 2017 | 10.3168/jds.2016-12446 |
| The acute-phase protein serum amyloid A3 is expressed in the bovine mammary gland and plays a role in host defence | Molenaar AJ et al. 2009 | 10.1080/13547500902730714 |
| The Antisecretory Factor in Plasma and Breast Milk in Breastfeeding Mothers-A Prospective Cohort Study in Sweden | Gustafsson A et al. 2018 | 10.3390/nu10091227 |
| The Effect of Lipopolysaccharide-Induced Experimental Bovine Mastitis on Clinical Parameters, Inflammatory Markers, and the Metabolome: A Kinetic Approach | Johnzon CF et al. 2018 | 10.3389/fimmu.2018.01487 |
| The major acute phase proteins of bovine milk in a commercial dairy herd | Thomas FC et al. 2015 | 10.1186/s12917-015-0533-3 |
| The production and characterization of anti-bovine CD14 monoclonal antibodies | Sohn EJ et al. 2004 | 10.1051/vetres:2004035 |
| The proteomic advantage: label-free quantification of proteins expressed in bovine milk during experimentally induced coliform mastitis | Boehmer JL et al. 2010 | 10.1016/j.vetimm.2010.10.004 |
| The relationship between the variants of the bovine MBL2 gene and milk production traits, mastitis, serum MBL-C levels and complement activity | Wang X et al. 2012 | 10.1016/j.vetimm.2012.06.017 |
| Three novel single-nucleotide polymorphisms of complement component 4 gene(C4A) in Chinese Holstein cattle and their associations with milk performance traits and CH50 | Yang Y et al. 2012 | 10.1016/j.vetimm.2011.11.010 |
| Use of milk amyloid A in the diagnosis of subclinical mastitis in dairy ewes | Miglio A et al. 2013 | 10.1017/S0022029913000484 |
| Use of serum amyloid A and milk amyloid A in the diagnosis of subclinical mastitis in dairy cows | Gerardi G et al. 2009 | 10.1017/S0022029909990057 |

**Table III.** Scopus search results

| **Title** | **Authors/Year** | **DOI** |
| --- | --- | --- |
| [C-reactive protein as a new parameter of mastitis] | Schrödl W et al. 1995 |  |
| 15-F2t-Isoprostane Concentrations and Oxidant Status in Lactating Dairy Cattle with Acute Coliform Mastitis | Mavangira, V et al. 2016 | 10.1111/jvim.13793 |
| A pilot study of acute phase proteins as indicators of bovine mastitis caused by different pathogens | Thomas, F.C et al. 2018 | 10.1016/j.rvsc.2018.06.015 |
| A proteomic perspective on the changes in milk proteins due to high somatic cell count | Zhang, L et al. 2015 | 10.3168/jds.2014-9279 |
| A proteomics-based identification of putative biomarkers for disease in bovine milk | van Altena et al. 2016 | 10.1016/j.vetimm.2016.04.005 |
| Advances in BHV1(IBR) research | Straub, O.C. 2001 |  |
| Association of polymorphism within LTF gene promoter with lactoferrin concentration in milk of Holstein cows | Zabolewicz T et al. 2014 | 10.1016/j.vetimm.2016.04.005 |
| Biomarker based detection of subclinical mastitis by liquid phase blocking elisa | Pranayapradhan et al. 2013 |  |
| Biosensor assay for determination of haptoglobin in bovine milk | Åkerstedt M et al. 2006 | 10.1017/S0022029906001774 |
| Bovine intra-mammary challenge with Streptococcus dysgalactiae spp. Dysgalactiae to explore the effect on the response of Complement activity | Maye, S et al. 2017 | 10.1016/j.vetimm.2016.04.005 |
| Changes in the content of whey proteins during lactation in cow's milk with a different somatic cells count | Sobczuk-Szul M et al. 2014 |  |
| Comparative diagnosis of infectious bacteria in bovine milk | Sedky D et al. 2020 | 10.17582/journal.jahp/2020/8.4.171.182 |
| Concentration of serum amyloid A and activity of ceruloplasmin in milk from cows with clinical and subclinical mastitis | Szczubiał M et al. 2008 | 10.2478/v10181-011-0149-x |
| Concentration of serum amyloid A and ceruloplasmin activity in milk from cows with subclinical mastitis caused by different pathogens | Szczubiał M et al. 2012 | 10.2478/v10181-011-0149-x |
| Concentrations of acute-phase proteins in milk from cows with clinical mastitis caused by different pathogens | Dalanezi FM et al. 2020 | 10.3390/pathogens9090706 |
| Determination of milk and blood concentrations of lipopolysaccharide-binding protein in cows with naturally acquired subclinical and clinical mastitis | Zeng R et al. 2009 | 10.3168/jds.2008-1636 |
| Development of an immunosensor assay for detection of haptoglobin in mastitic milk | Tan X et al. 2012 | 10.1111/j.1939-165X.2012.00468.x |
| Early post parturient changes in milk acute phase proteins | Thomas FC et al. 2016 | 10.1017/S0022029916000297 |
| Effects of induced energy deficiency on lactoferrin concentration in milk and the lactoferrin reaction of primary bovine mammary epithelial cells in vitro | Danowski, K et al. 2013 | 10.1111/j.1439-0396.2012.01305.x |
| Effects of intrauterine infusion of bacterial lipopolysaccharides on the mammary gland inflammatory response in goats | Purba FY et al. 2020 |  |
| Evaluation of a bovine cathelicidin ELISA for detecting mastitis in the dairy buffalo: Comparison with milk somatic cell count and bacteriological culture | Puggioni GMG et al. 2020 | 10.1016/j.rvsc.2019.11.009 |
| Evaluation of intramammary platelet concentrate efficacy as a subclinical mastitis treatment in dairy cows based on somatic cell count and milk amyloid A levels [Sütçü ıneklerde subklinik mastitis tedavisinde meme ıçi platelet konsantresi etkinliğinin somatik hücre sayımı ve süt amiloid a seviyeleri ıle değerlendirilmesi] | Evkuran DG et al. 2019 |  |
| Evaluation of milk cathelicidin for detection of bovine mastitis | Addis MF et al. 2016 | 10.3168/jds.2016-11407 |
| Evaluation of milk cathelicidin for detection of dairy sheep mastitis | Addis MF et al. 2016 | 10.3168/jds.2015-10293 |
| Expression of cathelicidins mRNA in the goat mammary gland and effect of the intramammary infusion of lipopolysaccharide on milk cathelicidin-2 concentration | Zhang GW et al. 2014 | 10.1016/j.vetmic.2014.01.029 |
| Facile construction of a molecularly imprinted polymer–based electrochemical sensor for the detection of milk amyloid A | Zhang Z et al. 2020 | 10.1016/j.vetmic.2014.01.029 |
| Factors affecting the lactoferrin concentration in bovine milk | Cheng JB et al. 2008 | 10.3168/jds.2007-0689 |
| Generation of an anti-NAGase single chain antibody and its application in a biosensor-based assay for the detection of NAGase in milk | Welbeck K et al. 2011 | 10.1016/j.jim.2010.09.019 |
| Gold nanoparticle size-dependent enhanced chemiluminescence for ultra-sensitive haptoglobin biomarker detection | Nirala, NR et al. 2019 | 10.3390/biom9080372 |
| [Haptoglobin and lactate dehydrogenase measurements in milk for the identification of subclinically diseased udder quarters](https://www.scopus.com/record/display.uri?eid=2-s2.0-34249695522&origin=resultslist&sort=plf-f&src=s&st1=&st2=&sid=0c46636c54b3545a6a58690e38be0dfb&sot=b&sdt=b&sl=47&s=TITLE-ABS-KEY+%28Haptoglobin+milk+mastitis+elisa%29&relpos=9&citeCnt=35&searchTerm=) | Hiss S et al. 2007 |  |
| Haptoglobin concentrations in blood and milk after endotoxin challenge and quantification of mammary Hp mRNA expression | Hiss S et al. 2004 | 10.3168/jds.S0022-0302(04)73516-X |
| Identification of lactoferrin-binding proteins in bovine mastitis-causing Streptococcus uberis | Fang W et al. 1999 | 10.1111/j.1574-6968.1999.tb13647.x |
| Immune-associated traits measured in milk of Holstein-Friesian cows as proxies for blood serum measurements | Denholm SJ et al. 2018 | 10.3168/jds.2018-14825 |
| Immunological responses of the lactating ovine udder following experimental challenge with Staphylococcus epidermidis | Winter P et al. 2002 |  |
| Immunosensing system for rapid multiplex detection of mastitis-causing pathogens in milk | Juronen D et al. 2018 | 10.1016/j.talanta.2017.10.043 |
| Increase of lactoferrin concentration in mastitic goat milk | Chen P et al. 2004 | 10.1292/jvms.66.345 |
| Interleukin-6 in quarter milk as a further prediction marker for bovine subclinical mastitis | Sakemi Y et al. 2011 | 10.1017/S0022029910000828 |
| [Interrelationship between somatic cell count and acute phase proteins in serum and milk of dairy cows](https://www.scopus.com/record/display.uri?eid=2-s2.0-42349099152&origin=resultslist&sort=plf-f&src=s&st1=&st2=&sid=0c46636c54b3545a6a58690e38be0dfb&sot=b&sdt=b&sl=47&s=TITLE-ABS-KEY+%28Haptoglobin+milk+mastitis+elisa%29&relpos=8&citeCnt=13&searchTerm=) | Kováč G et al. 2007 |  |
| [Kinetics of cells and cytokines during immune-mediated inflammation in the mammary gland of cows systemically immunized with Staphylococcus aureus α-toxin](https://www.scopus.com/record/display.uri?eid=2-s2.0-0033803061&origin=resultslist&sort=plf-f&src=s&st1=&st2=&sid=0c46636c54b3545a6a58690e38be0dfb&sot=b&sdt=b&sl=47&s=TITLE-ABS-KEY+%28Haptoglobin+milk+mastitis+elisa%29&relpos=12&citeCnt=45&searchTerm=) | Riollet C et al. 2000 | 10.1007/s000110050621 |
| Lactoferrin and IgG levels in ovine milk throughout lactation: Correlation with milk quality parameters | Navarro F et al. 2018 |  |
| Lactoferrin concentrations in bovine milk during involution of the mammary glands, with different bacteriological findings | Galfi A et al. 2016 |  |
| Lactoferrin concentrations in bovine milk prior to dry-off | Newman KA et al. 2009 | 10.1017/S0022029909990033 |
| Mastitis detection: current trends and future perspectives | Viguier C et al. 2009 | 10.1016/j.tibtech.2009.05.004 |
| Mid-infrared prediction of lactoferrin content in bovine milk: Potential indicator of mastitis | Soyeurt H et al. 2012 | 10.1017/S1751731112000791 |
| Milk cathelicidin and somatic cell counts in dairy goats along the course of lactation | Tedde V et al. 2019 | 10.1017/S0022029919000335 |
| Milk cytokines and subclinical breast inflammation in Tanzanian women: Effects of dietary red palm oil or sunflower oil supplementation | Filteau SM et al. 1999 | 10.1046/j.1365-2567.1999.00834.x |
| [Omic approaches to a better understanding of mastitis in dairy cows(Book Chapter)](https://www.scopus.com/record/display.uri?eid=2-s2.0-85042414725&origin=resultslist&sort=plf-f&src=s&st1=&st2=&sid=cd39eb29a6e4aa6ffd0130431b1eae48&sot=b&sdt=b&sl=49&s=TITLE-ABS-KEY+%28Amyloid+milk+mastitis+immunoassay%29&relpos=1&citeCnt=4&searchTerm=) | Mudaliar M et al. 2017 |  |
| Proteomic analysis of the temporal expression of bovine milk proteins during coliform mastitis and label-free relative quantification | Boehmer JL. et al 2010 | 10.3168/jds.2009-2526 |
| Purification of prostaglandin D synthase by ceramic- and size exclusion chromatography | Schlatterer JC et al. 2006 |  |
| Relationship between milk cathelicidin abundance and microbiologic culture in clinical mastitis | Addis MF et al. 2017 | 10.3168/jds.2016-12110 |
| Relationship of late lactation milk somatic cell count and cathelicidin with intramammary infection in small ruminants | Puggioni GMG et al. 2020 | 10.3390/pathogens9010037 |
| Serum amyloid A isoforms in serum and milk from cows with Staphylococcus aureus subclinical mastitis | Kovacevic-Filipovic M et al. 2012 | 10.1016/j.vetimm.2011.10.015 |
| Serum concentration and mRNA expression in milk somatic cells of toll-like receptor 2, toll-like receptor 4, and cytokines in dairy cows following intramammary inoculation with Escherichia coli | Ma JL et al. 2011 | 10.3168/jds.2011-4167 |
| Staphylococcus aureus lipoteichoic acid triggers inflammation in the lactating bovine mammary gland | Rainard P et al. 2008 |  |
| Susceptibility of sows to experimentally induced Escherichia coli mastitis. | Ross RF et al. 1983 |  |
| Technological interventions and advances in the diagnosis of intramammary infections in animals with emphasis on bovine population—a review | Chakraborty S et al. 2019 | 10.1080/01652176.2019.1642546 |
| Test characteristics of milk amyloid A ELISA, somatic cell count, and bacteriological culture for detection of intramammary pathogens that cause subclinical mastitis | Jaeger S et al. 2017 | 10.1016/j.vetimm.2011.10.015 |
| The antisecretory factor in plasma and breast milk in breastfeeding mothers—a prospective cohort study in Sweden | Gustafsson A et al. 2018 | 10.3390/nu10091227 |
| The diagnostic value of determination of positive and negative acute phase proteins in milk from dairy cows with subclinical mastitis | Shirazi-Beheshtiha SH et al. 2012 |  |
| The major acute phase proteins of bovine milk in a commercial dairy herd | Thomas FC et al. 2015 | 10.1186/s12917-015-0533-3 |
| The proteomic advantage: Label-free quantification of proteins expressed in bovine milk during experimentally induced coliform mastitis | Boehmer JL et al. 2010 | 10.1016/j.vetimm.2010.10.004 |
| The relationship between lactoferrin gene polymorphism and subclinical mastitis in awassi ewes | Alekish M et al. 2019 |  |
| Three novel single-nucleotide polymorphisms of complement component 4 gene(C4A) in Chinese Holstein cattle and their associations with milk performance traits and CH50 | Yang Y et al. 2012 | 10.1016/j.vetimm.2011.11.010 |
| Use of milk amyloid A in the diagnosis of subclinical mastitis in dairy ewes | Miglio A et al. 2013 | 10.1017/S0022029913000484 |
| Use of serum amyloid A and milk amyloid A in the diagnosis of subclinical mastitis in dairy cows | Gerardi G et al. 2009 | 10.1017/S0022029913000484 |

**Table IV.** WoS search results

| **Title** | **Authors/Year** | **DOI** |
| --- | --- | --- |
| 15-F-2t-Isoprostane Concentrations and Oxidant Status in Lactating Dairy Cattle with Acute Coliform Mastitis | Mavangira V et al. 2016 | 10.1111/jvim.13793 |
| A genome-wide association study for natural antibodies measured in blood of Canadian Holstein cows | de Klerk B et al. 2018 | 10.1186/s12864-018-5062-6 |
| A proteomics-based identification of putative biomarkers for disease in bovine milk | van Altena SEC et al. 2016 | 10.1016/j.vetimm.2016.04.005 |
| Acute phase response in lame crossbred dairy cattle | Bagga A et al. 2016 | 10.14202%2Fvetworld.2016.1204-1208 |
| Advances in BHV1(IBR) research | Straub OC 2001 |  |
| Assessment of milk quality trough microbiological an cytometric examination and determination of acute-phase proteins | Medvid V et al. 2011 |  |
| Association of polymorphism within LTF gene promoter with lactoferrin concentration in milk of Holstein cows | Zabolewicz T et al. 2014 | 10.2478/pjvs-2014-0094 |
| Binding of bovine lactoferrin to Streptococcus dysgalactiae subsp dysgalactiae isolated from cows with mastitis | Park HM et al. 2002 | 10.1111/j.1574-6968.2002.tb11057.x |
| Biosensor assay for determination of haptoglobin in bovine milk | Åkerstedt M et al. 2006 | 10.1017/S0022029906001774 |
| Bovine intra-mammary challenge with Streptococcus dysgalactiae spp. Dysgalactiae to explore the effect on the response of Complement activity | Maye S et al. 2017 | 10.1016/j.vetimm.2016.04.005 |
| Concentration of serum amyloid A and ceruloplasmin activity in milk from cows with subclinical mastitis caused by different, pathogens | Szczubial M et al. 2012 | 10.2478/v10181-011-0149-x |
| Crosstalk between coagulation and inflammation in mastitis and metritis in dairy cows | Bobowiec r et al. 2009 | 10.1556/AVet.57.2009.2.9 |
| Cytokine and acute phase protein gene expression in repeated liver biopsies of dairy cows with a lipopolysaccharide-induced mastitis | Vels L et al. 2009 | 10.3168/jds.2008-1209 |
| Detection of lactoferrin in bovine and goat milk by enzyme-linked immunosorbent assay | Chen PW et al. 2004 | 10.38212/2224-6614.2653 |
| Determination of milk and blood concentrations of lipopolysaccharide-binding protein in cows with naturally acquired subclinical and clinical mastitis | Zeng R et al. 2009 | 10.3168/jds.2008-1636 |
| Development and validation of an ELISA for the quantification of bovine ITIH4 in serum and milk | Soler L et al. 2019 |  |
| Development of an immunosensor assay for detection of haptoglobin in mastitic milk | Tan X et al. 2012 | 10.1111/j.1939-165X.2012.00468.x |
| Dietary-induced negative energy balance has minimal effects on innate immunity during a Streptococcus uberis mastitis challenge in dairy cows during midlactation | Moyes KM et al. 2009 | 10.3168/jds.2009-2170 |
| Effect of gestation length on the levels of five innate defence proteins in human milk | Broadhurst M et al. 2015 | 10.1016/j.earlhumdev.2014.11.006 |
| Effects of induced energy deficiency on lactoferrin concentration in milk and the lactoferrin reaction of primary bovine mammary epithelial cells in vitro | Danowski K et al. 2103 | 10.1111/j.1439-0396.2012.01305.x |
| Evaluation of a bovine cathelicidin ELISA for detecting mastitis in the dairy buffalo: Comparison with milk somatic cell count and bacteriological culture | Puggioni GMG et al. 2020 | 10.1016/j.rvsc.2019.11.009 |
| Evaluation of Intramammary Platelet Concentrate Efficacy as a Subclinical Mastitis Treatment in Dairy Cows Based on Somatic Cell Count and Milk Amyloid A Levels | Evkuran DG et al. 2019 | 10.9775/kvfd.2018.20982 |
| Evaluation of milk cathelicidin for detection of bovine mastitis | Addis MF et al. 2016 | 10.3168/jds.2016-11407 |
| Evaluation of milk cathelicidin for detection of dairy sheep mastitis | Addis MF et al. 2016 | [10.3168/jds.2015-10293](https://doi.org/10.3168/jds.2015-10293) |
| Expression of cathelicidins mRNA in the goat mammary gland and effect of the intramammary infusion of lipopolysaccharide on milk cathelicidin-2 concentration | Zhang GW et al. 2014 | 10.1016/j.vetmic.2014.01.029 |
| Facile construction of a molecularly imprinted polymer-based electrochemical sensor for the detection of milk amyloid A | Zhang ZR et al. 2020 | 10.1007/s00604-020-04619-7 |
| Factors affecting the lactoferrin concentration in bovine milk | ChengJB et al. 2008 | 10.3168/jds.2007-0689 |
| Generation of an anti-NAGase single chain antibody and its application in a biosensor-based assay for the detection of NAGase in milk | Welbeck K et al. 2011 | 10.1016/j.jim.2010.09.019 |
| Gold Nanoparticle Size-Dependent Enhanced Chemiluminescence for Ultra-Sensitive Haptoglobin Biomarker Detection | Nirala NR et al. 2019 | 10.3390/biom9080372 |
| Haptoglobin and lactate dehydrogenase measurements in milk for the identification of subclinically diseased udder quarters | Hiss S et al. 2007 | 10.17221/1879-vetmed |
| Haptoglobin concentrations in blood and milk after endotoxin challenge and quantification of mammary Hp mRNA expression | Hiss S et al. 2004 | 10.3390/biom9080372 |
| Identification of lactoferrin-binding proteins in bovine mastitis-causing Streptococcus uberis | Fang et al. 1999 | 10.1111/j.1574-6968.1999.tb13647.x |
| Immune-associated traits measured in milk of Holstein-Friesian cows as proxies for blood serum measurements | Denholm SJ et al. 2018 | 10.3168/jds.2018-14825 |
| Increase of lactoferrin concentration in mastitic goat milk | Chen PW et al. 2004 | 10.1292/jvms.66.345 |
| Influence of subclinical mastitis and intramammary infection by coagulase-negative staphylococci on the cow milk peptidome | Addis MF et al. 2020 |  |
| Interleukin-6 in quarter milk as a further prediction marker for bovine subclinical mastitis | Sakemi Y et al. 2011 | 10.1017/S0022029910000828 |
| Interrelationship between somatic cell count and acute phase proteins in serum and milk of dairy cows | Kováč G et al. 2007 | 10.2754/avb200776010051 |
| Kinetics of cells and cytokines during immune-mediated inflammation in the mammary gland of cows systemically immunized with Staphylococcus aureus alpha-toxin | Riollet C et al. 2000 | 10.1007/s000110050621 |
| Lactoferrin and IgG levels in ovine milk throughout lactation: Correlation with milk quality parameters | Navarro F et al. 2018 | 10.1016/j.smallrumres.2018.09.002 |
| Lactoferrin and Immunoglobulin G Concentration in Bovine Milk from Cows with Subclinical Mastitis during the Late Lactation Period | Galfi A et al. 2016 |  |
| Lactoferrin concentrations in bovine milk during involution of the mammary glands, with different bacteriological findings | Galfi A et al. 2016 |  |
| Lactoferrin concentrations in bovine milk prior to dry-off | Newman KA et al. 2009 | 10.1017/S0022029909990033 |
| Lactoferrin concentrations in goat milk throughout lactation | Hiss S et al. 2008 | 10.1016/j.smallrumres.2008.07.027 |
| MicroRNA Milk Exosomes: From Cellular Regulator to Genomic Marker | Cintio M et al. 2020 | 10.3390/ani10071126 |
| Mid-infrared prediction of lactoferrin content in bovine milk: potential indicator of mastitis | Soyeurt H et al. 2012 | 10.1017/S1751731112000791 |
| Milk cathelicidin and somatic cell counts in dairy goats along the course of lactation | Tedde V et al. 2019 | 10.1017/S0022029919000335 |
| Milk haptoglobin detection based on enhanced chemiluminescence of gold nanoparticles | Nirala NR et al. 2019 | 10.1016/j.talanta.2019.01.027 |
| Milk lactoferrin concentrations in anatolian buffaloes with and without subclinical mastitis | Ozenc E et al. 2019 |  |
| mRNA expression of immune factors and milk proteins in mammary tissue and milk cells and their concentration in milk during subclinical mastitis | Schmitz S et al. 2004 |  |
| Pilot study into milk haptoglobin as an indicator of udder health in heifers after calving | Simoes PBA et al. 2018 | 10.1016/j.rvsc.2017.05.024 |
| Plasma lactoferrin concentration measured by ELISA in healthy and diseased cows | Sato R et al. 2000 |  |
| Pro-inflammatory cytokine profile in dairy cows: consequences for new lactation | Trevisi E et al. 2015 |  |
| Proteomic analysis of the temporal expression of bovine milk proteins during coliform mastitis and label-free relative quantification | Boehmer JL et al. 2010 | 10.3168/jds.2009-2526 |
| Rapid biosensing of Staphylococcus aureus bacteria in milk | Peedel D et al. 2014 | 10.1039/c3ay42036a |
| Relationship between milk cathelicidin abundance and microbiologic culture in clinical mastitis | Addis MF et al. 2017 | 10.3168/jds.2016-12110 |
| Relationship of Late Lactation Milk Somatic Cell Count and Cathelicidin with Intramammary Infection in Small Ruminants | Puggioni GMG et al. 2020 | 10.3390/pathogens9010037 |
| Serum amyloid A isoforms in serum and milk from cows with Staphylococcus aureus subclinical mastitis | Kovacevic-Filipovic M et al. 2012 | 10.1016/j.vetimm.2011.10.015 |
| Serum and milk concentrations of oxidant and anti-oxidant markers in dairy cows affected with bloody milk | Ismail ZB et al. 2020 |  |
| Serum concentration and mRNA expression in milk somatic cells of toll-like receptor 2, toll-like receptor 4, and cytokines in dairy cows following intramammary inoculation with Escherichia coli | Ma JL et al. 2011 | 10.1016/j.vetimm.2011.10.015 |
| Serum haptoglobin-matrix metalloproteinase 9(Hp-MMP 9) complex as a biomarker of systemic inflammation in cattle | Bannikov GA et al. 2011 |  |
| Short communication: Production of antimicrobial peptide S100A8 in the goat mammary gland and effect of intramammary infusion of lipopolysaccharide on S100A8 concentration in milk | Purba FY et al. 2019 | 10.3168/jds.2018-15396 |
| Test characteristics of milk amyloid A ELISA, somatic cell count, and bacteriological culture for detection of intramammary pathogens that cause subclinical mastitis | Jaeger S et al. 2017 | 10.1016/j.vetimm.2011.10.015 |
| The Antisecretory Factor in Plasma and Breast Milk in Breastfeeding MothersA Prospective Cohort Study in Sweden | Gustafsson A et al. 2018 | 10.3390/nu10091227 |
| The major acute phase proteins of bovine milk in a commercial dairy herd | Thomas FC et al. 2015 | 10.1186/s12917-015-0533-3 |
| The proteomic advantage: Label-free quantification of proteins expressed in bovine milk during experimentally induced coliform mastitis | Boehmer JL et al. 2010 | 10.1016/j.vetimm.2010.10.004 |
| The relationship between lactoferrin gene polymorphism and subclinical mastitis in awassi ewes | Alekish M et al. 2019 | 10.1016/j.vetimm.2012.06.017 |
| Three novel single-nucleotide polymorphisms of complement component 4 gene(C4A) in Chinese Holstein cattle and their associations with milk performance traits and CH50 | Yang Y et al. 2012 | 10.1016/j.vetimm.2011.11.010 |
| Tumor necrosis factor-alpha and haptoglobin in the blood serum and mammary gland lymph from cows with acute clinical mastitis in comparison to healthy control animals | Hagen J et al. 2011 |  |
| Tumour necrosis factor-alpha(TNF-alpha) increases nuclear factor kappa B(NF kappa B) activity in and interleukin-8(IL-8) release from bovine mammary epithelial cells | Fitzgerald DC et al. 2007 | 10.1016/j.vetimm.2006.12.008 |
| Ultrasensitive haptoglobin biomarker detection based on amplified chemiluminescence of magnetite nanoparticles | Nirala NR et al. 2020 | 10.1186/s12951-019-0569-9 |
| Use of milk amyloid A in the diagnosis of subclinical mastitis in dairy ewes | Miglio A et al. 2013 | 10.1017/S0022029913000484 |
| Use of serum amyloid A and milk amyloid A in the diagnosis of subclinical mastitis in dairy cows | Gerardi G et al. 2009 | 10.1017/S0022029909990057 |

**Table V.** Scientific articles integrated by the expert reviewer

| **Title** | **Authors/Year** | **DOI** |
| --- | --- | --- |
| Acute phase proteins in milk in naturally acquired bovine mastitis caused by different pathogens | Pyörälä S et al. 2011 | 10.1136/vr.d1120. Epub 2011 May 9 |
| Acute phase proteins in serum and milk from dairy cows with clinical mastitis | Eckersall PD et al. 2001 | 10.1136/vr.148.2.35 |
| Acute phase proteins in the diagnosis of bovine subclinical mastitis | Shahabeddin S et al. 2009 | 10.1111/j.1939-165X.2009.00156.x |
| Acute phase response in two consecutive experimentally induced E. coli intramammary infections in dairy cows. | Suojala L et al. 2008 | 10.1186/1751-0147-50-18 |
| Changes in acute-phase proteins and cytokines in serum and milk whey from dairy cows with naturally occurring peracute mastitis caused by Klebsiella pneumoniae and the relationship to clinical outcome | Hisaeda K et al. 2011 | 10.1292/jvms.10-0403 |
| Characterization of Haptoglobin Isotype in Milk of Mastitis-Affected Cows. | Upadhyaya I et al. 2016 | 10.3390/vetsci3040029 |
| Haptoglobin and serum amyloid A in bulk tank milk in relation to raw milk quality | Åkerstedt M et al. 2009 | 10.1017/S0022029906002305 |
| Haptoglobin and serum amyloid A in milk from dairy cows with chronic sub-clinical mastitis. | Grönlund U et al. 2005 | 10.1051/vetres:2004063 |
| Haptoglobin and serum amyloid A in relation to the somatic cell count in quarter, cow composite and bulk tank milk samples. | Åkerstedt M et al. 2007 | 10.1017/S0022029906002305 |
| Interleukin-6 in quarter milk as a further prediction marker for bovine subclinical mastitis. | Sakemi Y et al. 2010 | 10.1017/S0022029910000828 |
| Milk amyloid A as a biomarker for diagnosis of subclinical mastitis in cattle | Hussein HA et al. 2018 | 10.14202/vetworld.2018.34-41 |
| Milk amyloid A: correlation with cellular indices of mammary inflammation in cows with normal and raised serum amyloid A | Mahony MCO et al. 2006 | 10.1016/j.rvsc.2005.05.005 |
| Milk lactoferrin in heifers: influence of health status and stage of lactation. | Chaneton L et al. 2013 | 10.3168/jds.2012-6028 |
| Natural variation in biomarkers indicating mastitis in healthy cows | Åkerstedt M et al. 2011 | 10.1017/S0022029910000786 |
| Serum amyloid A as a marker of cow֨ s mastitis caused by Streptococcus sp. | Bochniarz M et al. 2020 | 10.1016/j.cimid.2020.101498 |
| The value of the biomarkers cathelicidin, milk amyloid A, and haptoglobin to diagnose and classify clinical and subclinical mastitis | Wollowski L et al. 2021 | 10.3168/jds.2020-18539. Epub 2020 Dec 23 |

**Table VI.** Inclusion and exclusion criteria used in the selection phases

1. Population: ruminant species
2. Matrix: milk
3. Object: Protein biomarkers
4. Measurement technique: Immunoassay
5. Language: English
6. Document types to be excluded: reviews, case reports, reports, book chapters, editorials, letters

| **Title** | **Title** | **Abstract** | **Full text** |
| --- | --- | --- | --- |
| [C-reactive protein as a new parameter of mastitis] |  |  |  |
| 15-F-2t-Isoprostane Concentrations and Oxidant Status in Lactating Dairy Cattle with Acute Coliform Mastitis |  |  |  |
| A genome-wide association study for natural antibodies measured in blood of Canadian Holstein cows |  |  |  |
| A pilot study of acute phase proteins as indicators of bovine mastitis caused by different pathogens |  |  |  |
| A proteomic perspective on the changes in milk proteins due to high somatic cell count |  |  |  |
| A proteomics-based identification of putative biomarkers for disease in bovine milk |  |  |  |
| Acute phase response in lame crossbred dairy cattle |  |  |  |
| Acute phase proteins in milk in naturally acquired bovine mastitis caused by different pathogens |  |  |  |
| Acute phase proteins in serum and milk from dairy cows with clinical mastitis |  |  |  |
| Acute phase proteins in the diagnosis of bovine subclinical mastitis |  |  |  |
| Acute phase response in two consecutive experimentally induced E. coli intramammary infections in dairy cows. |  |  |  |
| Advances in BHV1(IBR) research |  |  |  |
| Assessment of milk quality trough microbiological an cytometric examination and determination of acute-phase proteins |  |  |  |
| Binding of bovine lactoferrin to Streptococcus dysgalactiae subsp dysgalactiae isolated from cows with mastitis |  |  |  |
| Biomarker based detection of subclinical mastitis by liquid phase blocking elisa |  |  |  |
| Biosensor assay for determination of haptoglobin in bovine milk |  |  |  |
| Bovine intra-mammary challenge with Streptococcus dysgalactiae spp. Dysgalactiae to explore the effect on the response of Complement activity |  |  |  |
| Cathelicidin production and release by mammary epithelial cells during infectious mastitis |  |  |  |
| Changes in acute-phase proteins and cytokines in serum and milk whey from dairy cows with naturally occurring peracute mastitis caused by Klebsiella pneumoniae and the relationship to clinical outcome |  |  |  |
| Changes in the content of whey proteins during lactation in cow's milk with a different somatic cells count |  |  |  |
| Characterization of Haptoglobin Isotype in Milk of Mastitis-Affected Cows. |  |  |  |
| Comparative diagnosis of infectious bacteria in bovine milk |  |  |  |
| Concentration of serum amyloid a and activity of ceruloplasmin in milk from cows with clinical and subclinical mastitis |  |  |  |
| Concentration of serum amyloid A and ceruloplasmin activity in milk from cows with subclinical mastitis caused by different pathogens |  |  |  |
| Concentrations of acute-phase proteins in milk from cows with clinical mastitis caused by different pathogens |  |  |  |
| Crosstalk between coagulation and inflammation in mastitis and metritis in dairy cows |  |  |  |
| Cytokine and acute phase protein gene expression in repeated liver biopsies of dairy cows with a lipopolysaccharide-induced mastitis |  |  |  |
| Detection of lactoferrin in bovine and goat milk by enzyme-linked immunosorbent assay |  |  |  |
| Determination of milk and blood concentrations of lipopolysaccharide-binding protein in cows with naturally acquired subclinical and clinical mastitis |  |  |  |
| Development and validation of an ELISA for the quantification of bovine ITIH4 in serum and milk |  |  |  |
| Development of an immunosensor assay for detection of haptoglobin in mastitic milk |  |  |  |
| Dietary-induced negative energy balance has minimal effects on innate immunity during a Streptococcus uberis mastitis challenge in dairy cows during midlactation |  |  |  |
| Dynamics of experimentally induced Staphylococcus epidermidis mastitis in East Friesian milk ewes |  |  |  |
| Early pathogenesis and inflammatory response in experimental bovine mastitis due to Streptococcus uberis |  |  |  |
| Early post parturient changes in milk acute phase proteins |  |  |  |
| Effect of gestation length on the levels of five innate defence proteins in human milk |  |  |  |
| Effect of intramammary infusion of tumour necrosis factor-alpha on milk protein composition and induction of acute-phase protein in the lactating cow |  |  |  |
| Effects of induced energy deficiency on lactoferrin concentration in milk and the lactoferrin reaction of primary bovine mammary epithelial cells in vitro |  |  |  |
| Effects of intrauterine infusion of bacterial lipopolysaccharides on the mammary gland inflammatory response in goats |  |  |  |
| Effects of lactoferrin and milk on adherence of Streptococcus uberis to bovine mammary epithelial cells |  |  |  |
| Elevated milk soluble CD14 in bovine mammary glands challenged with Escherichia coli lipopolysaccharide |  |  |  |
| Escherichia coli and Staphylococcus aureus elicit differential innate immune responses following intramammary infection |  |  |  |
| Evaluation of a bovine cathelicidin ELISA for detecting mastitis in the dairy buffalo: Comparison with milk somatic cell count and bacteriological culture |  |  |  |
| Evaluation of intramammary platelet concentrate efficacy as a subclinical mastitis treatment in dairy cows based on somatic cell count and milk amyloid A levels [Sütçü ıneklerde subklinik mastitis tedavisinde meme ıçi platelet konsantresi etkinliğinin somatik hücre sayımı ve süt amiloid a seviyeleri ıle değerlendirilmesi] |  |  |  |
| Evaluation of milk cathelicidin for detection of bovine mastitis |  |  |  |
| Evaluation of milk cathelicidin for detection of dairy sheep mastitis |  |  |  |
| Expression of cathelicidins mRNA in the goat mammary gland and effect of the intramammary infusion of lipopolysaccharide on milk cathelicidin-2 concentration |  |  |  |
| Expression of the peptidoglycan recognition protein, PGRP, in the lactating mammary gland |  |  |  |
| Facile construction of a molecularly imprinted polymer-based electrochemical sensor for the detection of milk amyloid A |  |  |  |
| Factors affecting the lactoferrin concentration in bovine milk |  |  |  |
| Factors associated with concentrations of select cytokine and acute phase proteins in dairy cows with naturally occurring clinical mastitis |  |  |  |
| Generation of an anti-NAGase single chain antibody and its application in a biosensor-based assay for the detection of NAGase in milk |  |  |  |
| Genetic variability of lactoferrin content estimated by mid-infrared spectrometry in bovine milk |  |  |  |
| Gold nanoparticle size-dependent enhanced chemiluminescence for ultra-sensitive haptoglobin biomarker detection |  |  |  |
| [Haptoglobin and lactate dehydrogenase measurements in milk for the identification of subclinically diseased udder quarters](https://www.scopus.com/record/display.uri?eid=2-s2.0-34249695522&origin=resultslist&sort=plf-f&src=s&st1=&st2=&sid=0c46636c54b3545a6a58690e38be0dfb&sot=b&sdt=b&sl=47&s=TITLE-ABS-KEY+%28Haptoglobin+milk+mastitis+elisa%29&relpos=9&citeCnt=35&searchTerm=) |  |  |  |
| Haptoglobin and serum amyloid A in bulk tank milk in relation to raw milk quality |  |  |  |
| Haptoglobin and serum amyloid A in milk from dairy cows with chronic sub-clinical mastitis. |  |  |  |
| Haptoglobin and serum amyloid A in relation to the somatic cell count in quarter, cow composite and bulk tank milk samples. |  |  |  |
| Haptoglobin concentrations in blood and milk after endotoxin challenge and quantification of mammary Hp mRNA expression |  |  |  |
| Identification of lactoferrin-binding proteins in bovine mastitis-causing Streptococcus uberis |  |  |  |
| Immune-associated traits measured in milk of Holstein-Friesian cows as proxies for blood serum measurements |  |  |  |
| Immunological responses of the lactating ovine udder following experimental challenge with Staphylococcus epidermidis |  |  |  |
| Immunosensing system for rapid multiplex detection of mastitis-causing pathogens in milk |  |  |  |
| Increase in milk metalloproteinase activity and vascular permeability in bovine endotoxin-induced and naturally occurring Escherichia coli mastitis |  |  |  |
| Increase of lactoferrin concentration in mastitic goat milk |  |  |  |
| Increased Epstein-Barr virus in breast milk occurs with subclinical mastitis and HIV shedding |  |  |  |
| Influence of bacterial factors on proliferation of bovine mammary epithelial cells |  |  |  |
| Influence of subclinical mastitis and intramammary infection by coagulase-negative staphylococci on the cow milk peptidome |  |  |  |
| Innate immune response in experimentally induced bovine intramammary infection with Staphylococcus simulans and S. epidermidis |  |  |  |
| Interleukin-6 in quarter milk as a further prediction marker for bovine subclinical mastitis |  |  |  |
| [Interrelationship between somatic cell count and acute phase proteins in serum and milk of dairy cows](https://www.scopus.com/record/display.uri?eid=2-s2.0-42349099152&origin=resultslist&sort=plf-f&src=s&st1=&st2=&sid=0c46636c54b3545a6a58690e38be0dfb&sot=b&sdt=b&sl=47&s=TITLE-ABS-KEY+%28Haptoglobin+milk+mastitis+elisa%29&relpos=8&citeCnt=13&searchTerm=) |  |  |  |
| Kinetics of cells and cytokines during immune-mediated inflammation in the mammary gland of cows systemically immunized with Staphylococcus aureus alpha-toxin |  |  |  |
| Kinetics of local and systemic isoforms of serum amyloid A in bovine mastitic milk |  |  |  |
| Lactoferrin and IgG levels in ovine milk throughout lactation: Correlation with milk quality parameters |  |  |  |
| Lactoferrin and Immunoglobulin G Concentration in Bovine Milk from Cows with Subclinical Mastitis during the Late Lactation Period |  |  |  |
| Lactoferrin concentrations in bovine milk during involution of the mammary glands, with different bacteriological findings |  |  |  |
| Lactoferrin concentrations in bovine milk prior to dry-off |  |  |  |
| Lactoferrin concentrations in goat milk throughout lactation |  |  |  |
| Low-level laser therapy attenuates LPS-induced rats mastitis by inhibiting polymorphonuclear neutrophil adhesion |  |  |  |
| Mastitis detection: current trends and future perspectives |  |  |  |
| Mastitis is associated with IL-6 levels and milk fat globule size in breast milk |  |  |  |
| MicroRNA Milk Exosomes: From Cellular Regulator to Genomic Marker |  |  |  |
| Mid-infrared prediction of lactoferrin content in bovine milk: potential indicator of mastitis |  |  |  |
| Milk amyloid A as a biomarker for diagnosis of subclinical mastitis in cattle |  |  |  |
| Milk amyloid A: correlation with cellular indices of mammary inflammation in cows with normal and raised serum amyloid A |  |  |  |
| Milk cathelicidin and somatic cell counts in dairy goats along the course of lactation |  |  |  |
| Milk cytokines and subclinical breast inflammation in Tanzanian women: effects of dietary red palm oil or sunflower oil supplementation |  |  |  |
| Milk haptoglobin detection based on enhanced chemiluminescence of gold nanoparticles |  |  |  |
| Milk lactoferrin concentrations in anatolian buffaloes with and without subclinical mastitis |  |  |  |
| Milk lactoferrin in heifers: influence of health status and stage of lactation. |  |  |  |
| Milk prostaglandins and electrical conductivity in bovine mastitis |  |  |  |
| mRNA expression of immune factors and milk proteins in mammary tissue and milk cells and their concentration in milk during subclinical mastitis |  |  |  |
| Natural variation in biomarkers indicating mastitis in healthy cows |  |  |  |
| [Omic approaches to a better understanding of mastitis in dairy cows( Book Chapter)](https://www.scopus.com/record/display.uri?eid=2-s2.0-85042414725&origin=resultslist&sort=plf-f&src=s&st1=&st2=&sid=cd39eb29a6e4aa6ffd0130431b1eae48&sot=b&sdt=b&sl=49&s=TITLE-ABS-KEY+%28Amyloid+milk+mastitis+immunoassay%29&relpos=1&citeCnt=4&searchTerm=) |  |  |  |
| Pilot study into milk haptoglobin as an indicator of udder health in heifers after calving |  |  |  |
| Plasma lactoferrin concentration measured by ELISA in healthy and diseased cows |  |  |  |
| Pro-inflammatory cytokine profile in dairy cows: consequences for new lactation |  |  |  |
| Proteomic analysis of the temporal expression of bovine milk proteins during coliform mastitis and label-free relative quantification |  |  |  |
| Proteomics and pathway analyses of the milk fat globule in sheep naturally infected by Mycoplasma agalactiae provide indications of the in vivo response of the mammary epithelium to bacterial infection |  |  |  |
| Purification of prostaglandin D synthase by ceramic- and size exclusion chromatography |  |  |  |
| Rapid biosensing of Staphylococcus aureus bacteria in milk |  |  |  |
| Relationship between milk cathelicidin abundance and microbiologic culture in clinical mastitis |  |  |  |
| Relationship between milk lactoferrin and etiological agent in the mastitic bovine mammary gland |  |  |  |
| Relationship of Late Lactation Milk Somatic Cell Count and Cathelicidin with Intramammary Infection in Small Ruminants |  |  |  |
| Serum amyloid A as an marker of cow֨ s mastitis caused by Streptococcus sp. |  |  |  |
| Serum amyloid A isoforms in serum and milk from cows with Staphylococcus aureus subclinical mastitis |  |  |  |
| Serum and milk concentrations of oxidant and anti-oxidant markers in dairy cows affected with bloody milk |  |  |  |
| Serum concentration and mRNA expression in milk somatic cells of toll-like receptor 2, toll-like receptor 4, and cytokines in dairy cows following intramammary inoculation with Escherichia coli |  |  |  |
| Serum C-reactive protein in dairy herds |  |  |  |
| Serum haptoglobin-matrix metalloproteinase 9(Hp-MMP 9) complex as a biomarker of systemic inflammation in cattle |  |  |  |
| Short communication: Production of antimicrobial peptide S100A8 in the goat mammary gland and effect of intramammary infusion of lipopolysaccharide on S100A8 concentration in milk |  |  |  |
| Staphylococcus aureus lipoteichoic acid triggers inflammation in the lactating bovine mammary gland |  |  |  |
| Susceptibility of sows to experimentally induced Escherichia coli mastitis |  |  |  |
| Technological interventions and advances in the diagnosis of intramammary infections in animals with emphasis on bovine population—a review |  |  |  |
| Test characteristics of milk amyloid A ELISA, somatic cell count, and bacteriological culture for detection of intramammary pathogens that cause subclinical mastitis |  |  |  |
| The acute-phase protein serum amyloid A3 is expressed in the bovine mammary gland and plays a role in host defence |  |  |  |
| The antisecretory factor in plasma and breast milk in breastfeeding mothers—a prospective cohort study in Sweden |  |  |  |
| The diagnostic value of determination of positive and negative acute phase proteins in milk from dairy cows with subclinical mastitis |  |  |  |
| The Effect of Lipopolysaccharide-Induced Experimental Bovine Mastitis on Clinical Parameters, Inflammatory Markers, and the Metabolome: A Kinetic Approach |  |  |  |
| The major acute phase proteins of bovine milk in a commercial dairy herd |  |  |  |
| The production and characterization of anti-bovine CD14 monoclonal antibodies |  |  |  |
| The proteomic advantage: label-free quantification of proteins expressed in bovine milk during experimentally induced coliform mastitis |  |  |  |
| The relationship between lactoferrin gene polymorphism and subclinical mastitis in awassi ewes |  |  |  |
| The relationship between the variants of the bovine MBL2 gene and milk production traits, mastitis, serum MBL-C levels and complement activity |  |  |  |
| The value of the biomarkers cathelicidin, milk amyloid A, and haptoglobin to diagnose and classify clinical and subclinical mastitis |  |  |  |
| Three novel single-nucleotide polymorphisms of complement component 4 gene(C4A) in Chinese Holstein cattle and their associations with milk performance traits and CH50 |  |  |  |
| Tumor necrosis factor-alpha and haptoglobin in the blood serum and mammary gland lymph from cows with acute clinical mastitis in comparison to healthy control animals |  |  |  |
| Tumour necrosis factor-alpha(TNF-alpha) increases nuclear factor kappa B(NF kappa B) activity in and interleukin-8(IL-8) release from bovine mammary epithelial cells |  |  |  |
| Ultrasensitive haptoglobin biomarker detection based on amplified chemiluminescence of magnetite nanoparticles |  |  |  |
| Use of milk amyloid A in the diagnosis of subclinical mastitis in dairy ewes |  |  |  |
| Use of serum amyloid A and milk amyloid A in the diagnosis of subclinical mastitis in dairy cows |  |  |  |

**Table VII.** Scientific papers not included in the qualitative analysis.

| **Author** | **Species** | **Reason for exclusion** |
| --- | --- | --- |
| Åkerstedt et al. 2006 | Cow | Single animal data |
| Åkerstedt et al. 2007 | Cow | Missing data |
| Åkerstedt et al. 2011 | Cow | Single animal data |
| Chaneton et al. 2008 | Cow | Missing data |
| Chaneton et al. 2013 | Cow | Missing data |
| Chen and Mao, 2004 | Goat | Type of samples (bulk) |
| Eckersall et al. 2001 | Cow | Unsuitable data * |
| Evkuran Dal et al. 2019 | Cow | Missing data |
| Grönlund et al. 2005 | Cow | Unsuitable data *-ATP for CMT? |
| Hiss et al. 2004 | Cow | Missing data |
| Hiss et al. 2008 | Goat | Aim of the study did not match the review question |
| Navarro et al. 2018 | Sheep | Missing data |
| Newman et al. 2009 | Cow | Missing data |
| Nirala and Shtenberg, 2019 | Cow | Single animal data |
| O’Mahony et al. 2006 | Cow | Missing data |
| Pedersen et al. 2003 | Cow | Aim of the study did not match the review question |
| Simões et al. 2018 | Cow | Aim of the study did not match the review question |
| Szczubiał et al. 2008 | Cow | No full text available |
| Tan et al. 2012 | Cow | Single animal data |
| Thomas et al. 2016 | Cow | Aim of the study did not match the review question |
| Thomas et al. 2018 | Cow | Samples identified as “all” (healthy status not specified) |
| Welbeck et al. 2001 | Human | No dairy ruminant species |
| Zhang et al. 2014 | Goat | Missing data |

**Table VIII. The 33 articles selected for the qualitative synthesis.**

| **Title** | **Authors/Year** | **DOI** |
| --- | --- | --- |
| Acute phase proteins in milk in naturally acquired bovine mastitis caused by different pathogens | Pyörälä S et al. 2011 | 10.1136/vr.d1120. Epub 2011 May 9 |
| Acute phase proteins in the diagnosis of bovine subclinical mastitis | Shahabeddin S et al. 2009 | 10.1111/j.1939-165X.2009.00156.x |
| Acute phase response in two consecutive experimentally induced E. coli intramammary infections in dairy cows. | Suojala L et al. 2008 | 10.1186/1751-0147-50-18 |
| Changes in the content of whey proteins during lactation in cow's milk with a different somatic cells count | Sobczuk-Szul M et al. 2014 |  |
| Concentration of serum amyloid A and ceruloplasmin activity in milk from cows with subclinical mastitis caused by different pathogens | Szczubiał M et al. 2012 | 10.2478/v10181-011-0149-x |
| Concentrations of acute-phase proteins in milk from cows with clinical mastitis caused by different pathogens | Dalanezi FM et al. 2020 | 10.3390/pathogens9090706 |
| Detection of lactoferrin in bovine and goat milk by enzyme-linked immunosorbent assay | Chen PW et al. 2004 | 10.38212/2224-6614.2653 |
| Determination of milk and blood concentrations of lipopolysaccharide-binding protein in cows with naturally acquired subclinical and clinical mastitis | Zeng R et al. 2009 | 10.3168/jds.2008-1636 |
| Evaluation of a bovine cathelicidin ELISA for detecting mastitis in the dairy buffalo: Comparison with milk somatic cell count and bacteriological culture | Puggioni GMG et al. 2020 | 10.1016/j.rvsc.2019.11.009 |
| Evaluation of milk cathelicidin for detection of bovine mastitis | Addis MF et al. 2016 | 10.3168/jds.2016-11407 |
| Evaluation of milk cathelicidin for detection of dairy sheep mastitis | Addis MF et al. 2016 | 10.3168/jds.2015-10293 |
| Factors affecting the lactoferrin concentration in bovine milk | Cheng JB et al. 2008 | 10.3168/jds.2007-0689 |
| Factors associated with concentrations of select cytokine and acute phase proteins in dairy cows with naturally occurring clinical mastitis | Wenz JR et al. 2010 | 10.3168/jds.2009-2819 |
| [Haptoglobin and lactate dehydrogenase measurements in milk for the identification of subclinically diseased udder quarters](https://www.scopus.com/record/display.uri?eid=2-s2.0-34249695522&origin=resultslist&sort=plf-f&src=s&st1=&st2=&sid=0c46636c54b3545a6a58690e38be0dfb&sot=b&sdt=b&sl=47&s=TITLE-ABS-KEY+%28Haptoglobin+milk+mastitis+elisa%29&relpos=9&citeCnt=35&searchTerm=) | Hiss S et al. 2007 | 10.17221/1879-VETMED |
| Haptoglobin and serum amyloid A in bulk tank milk in relation to raw milk quality | Åkerstedt M et al. 2009 | 10.1017/S0022029906002305 |
| Immunological responses of the lactating ovine udder following experimental challenge with Staphylococcus epidermidis | Winter P et al. 2002 |  |
| Increase of lactoferrin concentration in mastitic goat milk | Chen PW et al. 2004 | 10.1292/jvms.66.345 |
| Interleukin-6 in quarter milk as a further prediction marker for bovine subclinical mastitis | Sakemi Yet al. 2011 | 10.1017/S0022029910000828 |
| Interrelationship between somatic cell count and acute phase proteins in serum and milk of dairy cows | Kováč G et al. 2007 | 10.2754/avb200776010051 |
| Lactoferrin and Immunoglobulin G Concentration in Bovine Milk from Cows with Subclinical Mastitis during the Late Lactation Period | Galfi A et al. 2016 |  |
| Lactoferrin concentrations in bovine milk during involution of the mammary glands, with different bacteriological findings | Galfi A et al. 2016 |  |
| Milk amyloid A as a biomarker for diagnosis of subclinical mastitis in cattle | Hussein HA et al. 2018 | 10.14202/vetworld.2018.34-41 |
| Milk cathelicidin and somatic cell counts in dairy goats along the course of lactation | Tedde V et al. 2019 | 10.1017/S0022029919000335 |
| Milk lactoferrin concentrations in anatolian buffaloes with and without subclinical mastitis | Ozenc E et al. 2019 |  |
| Relationship between milk cathelicidin abundance and microbiologic culture in clinical mastitis | Addis MF et al. 2017 | 10.3168/jds.2016-12110 |
| Relationship of Late Lactation Milk Somatic Cell Count and Cathelicidin with Intramammary Infection in Small Ruminants | Puggioni GMG et al. 2020 | 10.3390/pathogens9010037 |
| Serum amyloid A as an marker of cow֨ s mastitis caused by Streptococcus sp. | Bochniarz M et al. 2020 | 10.1016/j.cimid.2020.101498 |
| Serum amyloid A isoforms in serum and milk from cows with Staphylococcus aureus subclinical mastitis | Kovačević-Filipović M et al. 2003 | 10.1016/j.vetimm.2011.10.015 |
| Test characteristics of milk amyloid A ELISA, somatic cell count, and bacteriological culture for detection of intramammary pathogens that cause subclinical mastitis | Jaeger S et al. 2017 | 10.3168/jds.2016-12446 |
| The diagnostic value of determination of positive and negative acute phase proteins in milk from dairy cows with subclinical mastitis | Shirazi-Beheshtiha SH et al. 2012 |  |
| The major acute phase proteins of bovine milk in a commercial dairy herd | Thomas FC et al. 2015 | 10.1186/s12917-015-0533-3 |
| The value of the biomarkers cathelicidin, milk amyloid A, and haptoglobin to diagnose and classify clinical and subclinical mastitis | Wollowski L et al. 2021 | 10.3168/jds.2020-18539. Epub 2020 Dec 23 |
| Use of milk amyloid A in the diagnosis of subclinical mastitis in dairy ewes | Miglio A et al. 2013 | 10.1017/S0022029913000484 |
| Use of serum amyloid A and milk amyloid A in the diagnosis of subclinical mastitis in dairy cows | Gerardi G et al. 2009 | 10.1017/S0022029909990057 |

**Table IX** Bias assessment of eligible records for qualitative analysis

| **Scientific article** | **Animal Selection** | **Index**  **Test** | **Reference Standard** | **Flow and Timing** |
| --- | --- | --- | --- | --- |
| Addis et al. 2016a | Low | Unclear | Unclear | Low |
| Addis et al. 2016b | Low | Low | Unclear | Low |
| Addis et al. 2017 | High | High | Low | Low |
| Åkerstedt et al. 2009 | Low | High | High | Low |
| Bochniarz et al. 2020 | Low | High | Low | Unclear |
| Chen et al. 2004 | Low | High | High | Low |
| Cheng et al. 2008 | High | High | Low | High |
| Dalanezi et al. 2020 | Low | High | High | Low |
| Galfi et al. 2016a | High | High | High | Low |
| Galfi et al. 2016b | Low | High | High | Low |
| Gerardi et al. 2009 | High | High | Low | High |
| Hiss et al. 2007 | High | High | Low | Low |
| Hussein et al. 2018 | High | High | Low | High |
| Jaeger et al. 2017 | Low | High | Low | High |
| Kováč et al. 2007 | High | High | Low | High |
| Kovačević-Filipović et al. 2012 | Low | High | Low | Low |
| Miglio et al. 2013 | Low | High | Low | High |
| Özenç et al. 2019 | High | High | Low | High |
| Puggioni et al. 2020a | Low | Unclear | Low | Low |
| Puggioni et al. 2020b | Low | Unclear | Low | Low |
| Pyorala et al. 2011 | Low | High | High | Low |
| Safi et al. 2009 | High | High | Low | High |
| Sakemi et al. 2011 | Low | High | Low | High |
| Shirazi-Beheshtiha et al. 2011 | Low | High | Low | Low |
| Sobczuk-Szul et al. 2014 | High | High | High | Low |
| Suojala et al. 2008 | High | High | Low | Low |
| Szczubiał et al. 2012 | High | High | Low | Low |
| Tedde et al. 2019 | Low | High | Low | Low |
| Thomas et al. 2015 | Low | High | High | Low |
| Wenz et al. 2010 | High | High | Low | High |
| Winter and Colditz 2002 | High | High | High | Low |
| Wollowski et al. 2021 | High | High | Low | High |
| Zeng et al. 2009 | Low | High | Low | Low |

**Table X** Applicability of eligible records for qualitative analysis

| **Scientific article** | **Animal Selection** | **Index Test** | **Reference Standard** |
| --- | --- | --- | --- |
| Addis et al. 2016a | Low | Low | Low |
| Addis et al. 2016b | Low | Low | Low |
| Addis et al. 2017 | Low | Low | Low |
| Åkerstedt et al. 2009 | Low | Low | Low |
| Bochniarz et al. 2020 | Low | Low | Low |
| Chen et al. 2004 | Low | Low | Low |
| Cheng et al. 2008 | High | High | Low |
| Dalanezi et al. 2020 | Low | High | Low |
| Galfi et al. 2016a | High | High | Low |
| Galfi et al. 2016b | Low | High | High |
| Gerardi et al. 2009 | Low | Low | Low |
| Hiss et al. 2007 | Low | Low | Low |
| Hussein et al. 2018 | Low | Low | Low |
| Jaeger et al. 2017 | Low | Low | Low |
| Kováč et al. 2007 | Low | Low | Low |
| Kovačević-Filipović et al. 2012 | Low | Low | Low |
| Miglio et al. 2013 | Low | Low | Low |
| Özenç et al. 2019 | Low | Low | Low |
| Puggioni et al. 2020a | Low | Low | Low |
| Puggioni et al. 2020b | Low | Low | Low |
| Pyörälä et al. 2011 | Low | Low | High |
| Safi et al. 2009 | Low | Low | Low |
| Sakemi et al. 2011 | Low | Low | Low |
| Shirazi-Beheshtiha et al. 2011 | Low | Low | Low |
| Sobczuk-Szul et al. 2014 | High | Unclear | Low |
| Suojala et al. 2008 | Low | Unclear | Low |
| Szczubiał et al. 2012 | Low | Low | Low |
| Tedde et al. 2019 | Low | High | Low |
| Thomas et al. 2015 | Low | Low | Low |
| Wenz et al. 2010 | Low | Low | Low |
| Winter and Colditz 2002 | High | Low | Low |
| Wollowski et al. 2021 | Low | Low | Low |
| Zeng et al. 2009 | Low | Unclear | Low |
